# Selective oxidation of active site aromatic residues in engineered Cu proteins†

**DOI:** 10.1039/d4sc06667g

**Published:** 2024-11-18

**Authors:** Kylie S. Uyeda, Alec H. Follmer, A. S. Borovik

**Affiliations:** a Department of Chemistry, University of California–Irvine Irvine CA 92697 USA afollmer@uci.edu aborovik@uci.edu

## Abstract

Recent studies have revealed critical roles for the local environments surrounding metallocofactors, such as the newly identified Cu_D_ site in particulate methane monooxygenases (pMMOs) and the second sphere aromatic residues in lytic polysaccharide monooxygenases (LPMOs), implicated in the protection against oxidative damage. However, these features are subjects of continued debate. Our work utilizes biotin–streptavidin (Sav) technology to develop artificial metalloproteins (ArMs) that mimic the active sites of natural copper metalloenzymes. By engineering ArMs with aromatic residues within their secondary coordination spheres, we systematically investigate the influence of these residues on Cu reactivity and oxidant activation. We demonstrate that the placement and orientation of tyrosine relative to the Cu cofactor critically affect the oxidation outcomes upon exposure to hydrogen peroxide. A key finding is the interplay between the coordination of an active site asparagine and the incorporation of aromatic residues proximal to the artificial Cu cofactor, which are the only variants where oxidation of an engineered residues is observed. These findings underscore the importance of the secondary coordination sphere in modulating Cu center reactivity, suggest a role for amide coordination in C–H bond activation by pMMOs, and potential inactivation pathways in natural copper enzymes like LPMOs.

## Abstract 2

Engineered metalloproteins that mimic the active sites of natural copper metalloenzymes are used to systematically investigate the influence of aromatic residues within the secondary coordination sphere on reactivity.

## Graphical abstract

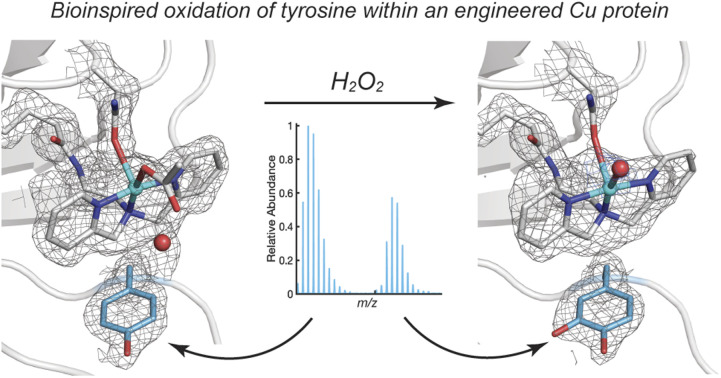

## Introduction

**Fig. 1:**
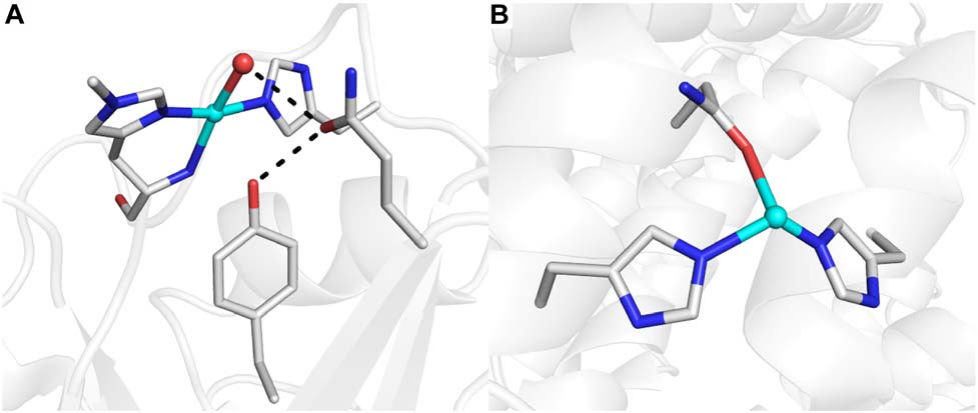
Molecular structures of the LPMO active site (A, PDB: 5ACH) and the Cu_D_ site of pMMO (B, PDB: 7S4H). H-bonding interactions are denoted by black dashed lines.

The development of synthetic methods for the selective functionalization of C–H bonds remains a major challenge in modern chemistry.^1,2^ Nature often utilizes metalloenzymes to facilitate such selective reactivity in oxidative transformations using O_2_ or H_2_O_2_ as primary oxidants, thus inspiring substantial efforts towards understanding their mechanisms and characterizing the intermediates involved during turnover.^3^ Insights from structural biology, biophysics, and synthetic model systems have demonstrated that effective C–H bond activation by metalloenzymes requires contributions from many properties associated with the confinement of metallocofactor(s) within the protein scaffold. The ligands bound to the metal ion(s) that comprise the primary coordination sphere are crucial for function. For example, in lytic polysaccharide monooxygenases (LPMOs) that degrade cellulose, the Cu center is ligated by a conserved set of donors, termed ‘the histidine brace’ (Fig. 1A), while in particulate methane monooxygenase (pMMO), the Cu_D_ site shows a unique coordination by a nearby asparagine residue (Fig. 1B).^4,5^ While the primary coordination has been the focus of many studies, it is now recognized that the local environment, the volume of space that surrounds a metallocofactor and includes the secondary coordination sphere, is also crucial for maintaining function.^6–10^ In LPMOs, aromatic residues positioned 3–5 Å from the Cu center are suggested to protect the Cu cofactor from oxidative damage during reactions with H_2_O_2_.^11–15^ In variants of LPMO where the nearby residue is a tyrosine, interaction with a nearby glutamine residue in the secondary sphere *via* a hydrogen bond (H-bond) is crucial for reactivity (Fig. 1A).^16^ These enzymes excel in functionalizing strong aliphatic bonds (∼100 kcal mol^−1^) under ambient conditions, a function that is difficult to duplicate in synthetic systems.^3^ One possible cause for these differences is the inability of synthetic systems to regulate local environments, such as placement of key amino acid residues like tyrosines proximal to a reactive metal species. Engineering artificial metalloenzymes (ArMs) offers an approach to examine the effects of local environments by providing a way to control individual structural components to systematically study their properties that lead to function.

Our group develops artificial metalloproteins (ArMs) using biotin–streptavidin (Sav) technology to mimic natural metalloenzyme active sites to characterize otherwise elusive intermediates and study the effects of the secondary coordination sphere on metal ion(s) reactivity. This approach leverages the strong binding affinity between biotinylated metal complexes and the Sav host to confine synthetic metal cofactors within a protein scaffold. In previous work, we prepared and characterized ArMs exhibiting biotinylated Cu cofactors (denoted as cofactors) as semi-synthetic models of both cupredoxins^17^ and the intermediate states of Cu metalloenzymes by stabilizing Cu^II^–hydroperoxido species.^6,18^ Therefore, we reasoned that we could engineer artificial Cu proteins with aromatic residues proximal to the Cu center to emulate aspects of LPMO active sites and understand the influence of these residues within the secondary coordination sphere on the activation of oxidants (Fig. S1†). The resulting Cu ArMs oxidize the installed aromatic residues, but the oxidation is dependent on the orientation of the tyrosine relative to the Cu cofactor. Moreover, we identify a new amide coordination to our Cu centers that emerges because of tyrosine incorporation and suggests an interplay between the two active site residues that is implicated in our ArMs reactivity. These findings highlight the importance of considering the composition and arrangement of residues within the secondary coordination sphere in engineering ArMs. Moreover, these results suggest one possible outcome for LPMO inactivation upon reacting with peroxide.

## Results and discussion

### Design considerations and preparations of ArMs

In LPMOs, hydrogen peroxide has been shown to enable catalytic activity in place of O_2_ and a reductant, although this reaction leads to significant protein degradation.^13,19^ Regardless of the oxidant, most mechanistic proposals invoke a Cu^II^–OOH species as a key intermediate.^11,13,19,20^ Our group has shown the ability to utilize ArMs to stabilize and structurally characterize similar Cu^II^–OOH species through the incorporation of H-bond networks within the secondary sphere.^18^ Specifically, incorporation of Cu^II^ ions into a biotinylated ligand (Scheme S1†) enabled the positioning of the metal center in close proximity to Sav residue S112, a site that is commonly employed for engineering ArMs with Sav.^17,21,22^ We reasoned that a tyrosine mutation at position S112 would be close enough to our Cu cofactors to influence the structure and reactivity but would not be oriented to participate in direct coordination. To prepare this type of Cu ArM, a triple mutant variant of Sav, referred to as 2XM-S112Y-Sav, was engineered; this mutant contains S112Y and two additional mutations, K121A and E101Q, which we have found to prevent unwanted interactions with our artificial metallocofactors (Fig. S2, S3 and Table S1†).^21^ We also utilized Cu^II^-biot-et-dpa as our cofactor to assemble our ArMs, where (biot-et-dpa) is bis(2-pyridylmethyl)amine.

### Structural characterization of [Cu^II^-biot-et-dpa⊂2XM-S112Y-Sav]

**Fig. 2:**
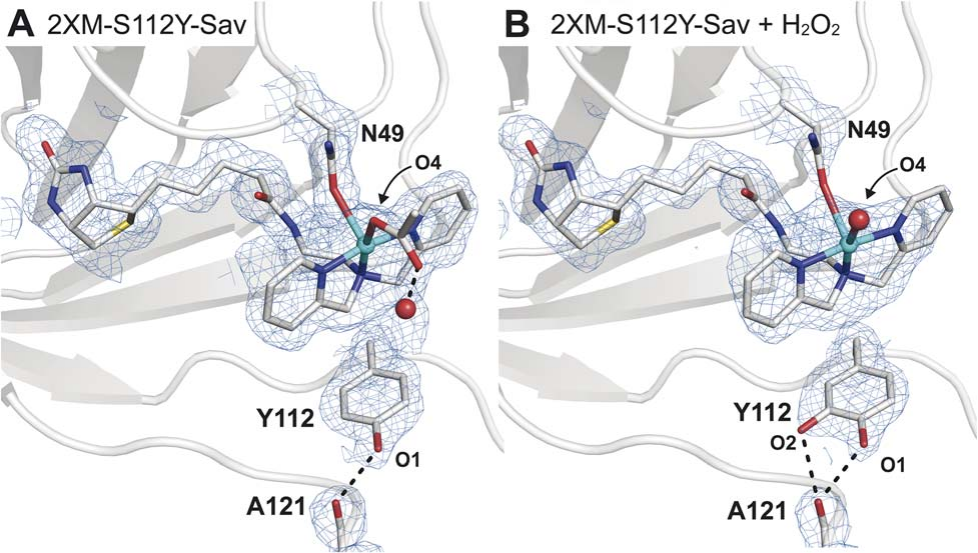
Molecular structures of 1 before (A, PDB: 9CSU) and after (B, PDB: 9CSV) the addition of H_2_O_2_. The 2*F*_o_ − *F*_c_ electron density map (blue mesh, contoured at 1*σ*) is highlighted in (B) with same labelling as in (A). In the molecular models of 1, N49 was found in two rotameric states, where the occupancy of the bound state is consistent with the Cu occupancy (∼65%) in the absence of H_2_O_2_. In the presence of H_2_O_2_, the occupancy of this rotamer is reduced to 51%, or roughly two-thirds of the refined Cu occupancy (74%). Only the N49 rotamer nearest to the Cu center is depicted for clarity. Selected bond lengths for 1 (1 + H_2_O_2_): Cu–N_avg_ = 2.0 Å (2.1 Å); Cu–O_N49_ = 2.5 Å (2.4 Å); in 1 Cu–O4_OAc(W)_ = 2.3 Å (2.4 Å) (Fig. S5 and Table S3†). The Cu–O_W_ distance in 1 is 2.9 Å. H-bonds are drawn as black dashed lines. Copper is colored in cyan, oxygen in red, nitrogen in blue, sulphur in yellow, and carbon in grey.

Single crystals of [Cu^II^-biot-et-dpa⊂2XM-S112Y-Sav] (1) were obtained *via* a soaking method described previously^17,18^ and diffracted to a 1.6 Å resolution (Fig. 2A and Table S2†). Determination of the molecular structure of 1 revealed a 5-coordinate, square pyramidal Cu^II^ cofactor with the dpa ligand coordinated in a meridional fashion (Fig. 2A and Table S2†). One of the remaining coordination sites is occupied by an O atom donor from an acetate ion originating from our crystallization conditions and bound in a κ^1^-manner – this ligand is also H-bonded to a water molecule within the secondary coordination sphere. The final coordination site is occupied by an O/N donor from the N49 sidechain, which undergoes a conformational change to produce a previously unobserved rotamer (Fig. 2 and S4B†). Although protein crystallography cannot unambiguously discern the identity of the coordinating atom of the N49 sidechain (*i.e.*, either O or N), we modelled the bound state as O-bound based on the pH of our crystallization conditions (pH = 6), which was below the p*K*_a_ of the amide.

Comparison of the structure of 1 to that of the analogous Cu ArM without the S112Y mutation ([Cu^II^-biot-et-dpa⊂2XM-Sav], 2) shows how the incorporation of the S112Y mutation both shifts the position of the copper center and changes the orientation preferences of the metallocofactor (Fig. S5†). Two distinct copper positions are observed in the anomalous electron difference density maps of 2 (Fig. S5A†), and only the dominant conformation of the ligand (refined Cu occupancy of 0.66) can be resolved due to overlap between the two. This result also suggests that the incorporation of tyrosine at position 112 in 1 induces a preferential orientation on the cofactor and shifts the Cu position by 0.6 Å towards N49 compared to in 2. The sterically driven change brings the cofactor in proximity for coordination to N49 in 1 (Fig. S5C and D†).

Unlike in our previous Cu-ArMs,^18^ treatment of 1 with hydrogen peroxide did not result in the observation of a Cu^II^–OOH adduct. However, incubation of single crystals of 1 with 1 mM H_2_O_2_ at pH = 6 for ∼10 min revealed a striking result. Specifically, we observed a small amount of positive difference density in the *F*_o_ − *F*_c_ electron density map that suggested an additional atom covalently bonded to S112Y (Fig. S7†). This observation is consistent with the oxidation of S112Y to yield a catechol-like species that formed bifurcated H-bonds with the backbone carbonyl of residue 121 (Fig. 2B); alternatively, the difference in density may be explained by the tyrosine residue adopting two conformations. Models of the latter possibility resulted in a structure with a negligible change in crystallographic refinement statistics, suggesting that both molecular models may be plausible and that additional studies are required to distinguish which possibility is correct (Fig. S8†).

### Evaluation of intact protein modification by LC-MS

**Fig. 3:**
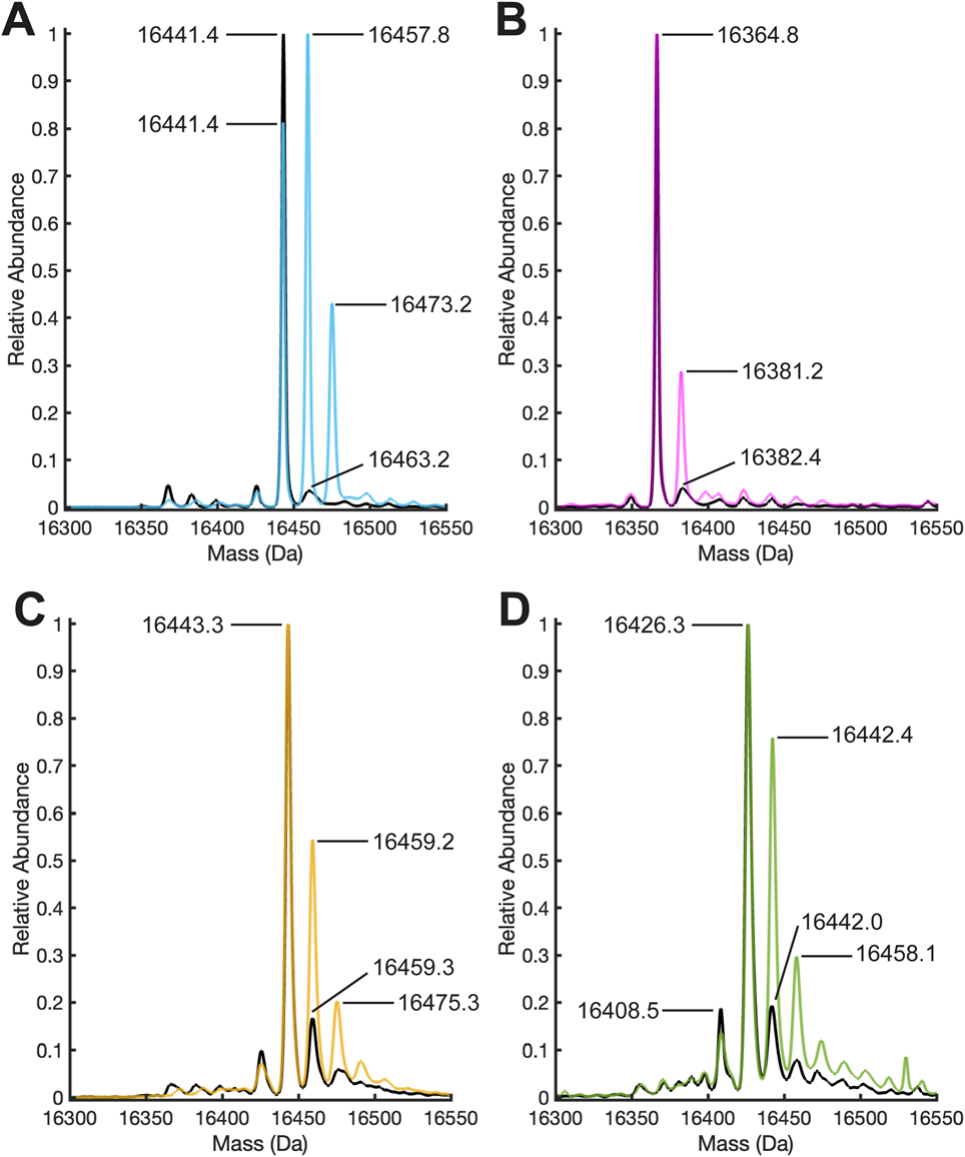
Deconvoluted ESI TOF positive ion mode mass spectra of (A) 1 before (black) and after (blue) the addition of 10 equivalents of H_2_O_2_ and incubation for 1 hour. (B) Deconvoluted mass spectra of 2 before (black) and after (pink) the addition of 10 equivalents of H_2_O_2_ and incubation for 1 hour. (C) Deconvoluted mass spectra of 3 before (black) and after (yellow) the addition of 10 equivalents of H_2_O_2_ and incubation for 1 hour. (D) Deconvoluted mass spectra of 4 before (black) and after (green) the addition of 10 equivalents of H_2_O_2_ and incubation for 1 hour.

To further evaluate what caused the change in the electron density maps of 1 upon treatment with H_2_O_2_, we performed liquid chromatography high resolution electrospray ionization quadrupole time-of-flight mass spectrometry (LC-HR-ESI-Q-TOF MS) in combination with enzymatic digestion (Tables S4 and S5†). The deconvoluted ESI mass spectra of 1 before and after treatment with H_2_O_2_ revealed significant oxidation of the monomers of 2XM-S112Y-Sav (16 441.4 *m*/*z*), as evidenced by the growth of two peaks in the peroxide-treated mass spectra at +16 and +32 *m*/*z*, corresponding to the singly and doubly oxidized species (Fig. 3a, S9, and S10†). Notice that the relative abundance of the *m*/*z* peak consistent with the singly oxidized species increases and replaces that of the unoxidized species as the most abundant. These results contrast with those obtained with 2, where in the absence of tyrosine, only a small amount of oxidation occurs (new peak at 16 381.2 *m*/*z*) and the unoxidized peak (16 364.8 *m*/*z*) remains the most abundant. As a control, we treated both 2XM-Sav and 2XM-S112Y-Sav in their apo forms and when bound to the biotinylated ligand with H_2_O_2_. Both apo proteins undergo non-specific oxidation, which decreases when the proteins are bound to the biotinylated ligand (see ESI for details and Fig. S11–15†). Upon reaction of 1 with H_2_^18^O_2_, a +17.6 *m*/*z* shift of the monomeric mass was observed, consistent with the incorporation of ^18^O atom (see ESI and Fig. S16†).

## Site of oxidation identified by trypsin digestion and LC-MS/MS

**Fig. 4:**
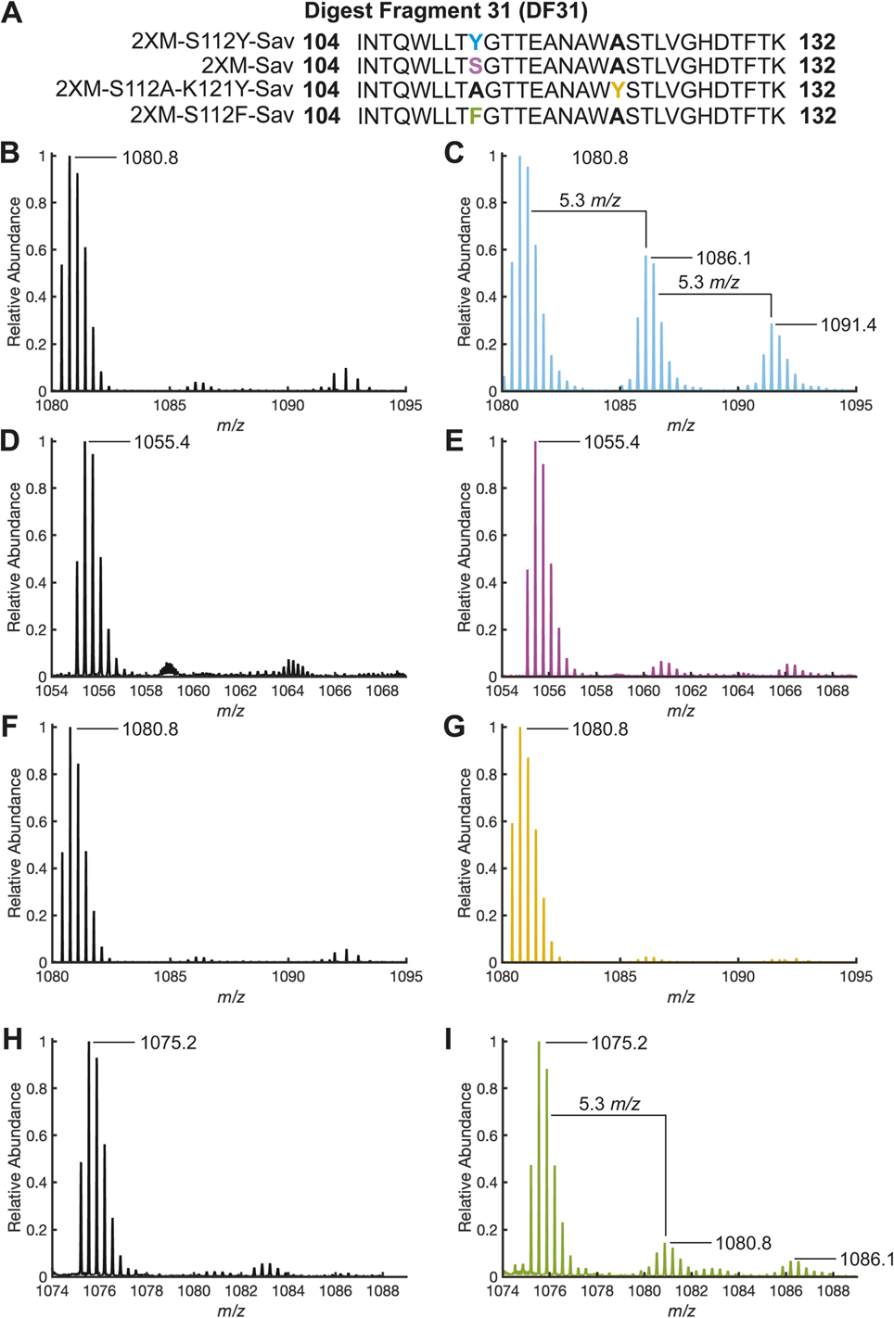
(A) Digest fragment 31 sequences of variant 1, 2, 3 and 4. ESI-TOF positive-ion mode mass spectra [DF31]^3+^ resulting from tryptic digests: 1 (B) before and (C) after the addition of 10 equivalents of H_2_O_2_ and incubation for 1 hour. 2 (D) before and (E) after the addition of 10 equivalents of H_2_O_2_ and incubation for 1 hour. 3 (F) before and (G) after the addition of 10 equivalents of H_2_O_2_ and incubation for 1 hour. 4 (H) before and (I) after the addition of 10 equivalents of H_2_O_2_ and incubation for 1 hour. Mass spectra were produced from the integration of LC peaks containing both unoxidized and oxidized DF31 (retention time: 2.85–3.10 min, Fig. S18–S20 and S27†).

While whole-protein MS provided support for the oxidation of the Sav variant, enzymatic digestion of the proteins with trypsin and analysis of the resulting digest fragments (DF) with LC-ESI-MS/MS revealed the location of oxidation within the Sav variants. Among the 44 possible DFs observed by this method (Table S6†), the most abundant modified DF (DF31) extends from residue 104 to 132 and contains positions 112 and 121. Upon reaction of 1 with H_2_O_2_, the mass spectrum of the triply charged ion of DF31 ([DF31]^3+^, 1080.8 *m*/*z*, retention time 3.07 min) exhibited two additional peaks that are shifted by 5.3 *m*/*z* (retention time 2.87 min) and 10.6 *m*/*z* (retention time 2.97 min) corresponding to single and double oxidation of DF31 (Fig. 4). The chromatogram was integrated over this entire range from 2.85 to 3.12 min to capture the relative abundance of both the unoxidized and oxidized DF31 species for further processing (Fig. S18 and S19†). Furthermore, MS/MS secondary fragmentation analysis of DF31 confirmed the oxidation of S112Y (Fig. S17, Table S7 and see ESI† for details). DF31 has other residues known to be susceptible to oxidation by H_2_O_2_, including one histidine (H127) and two tryptophan (W120 and W108) residues. However, these residues have centroid distances >9.6 Å from Cu, making them less likely to be modified than Y112, which sits 6.5 Å from Cu, but they may be the location of additional oxidation events (Fig. S21†). Moreover, the electron density maps of our structures do not exhibit significant difference density or suggest modification of these positions upon exposure to H_2_O_2_ (Fig. S22†).

Upon reaction of 2 with H_2_O_2_, we could identify the triply charged DF31 ([DF31^3+^], 1055.5 *m*/*z*, retention time 3.1 min), and a relatively small amount of the singly and doubly oxidized species, though their relative abundance is significantly less than in 1 (Fig. 4). Again, the chromatograms were integrated over the range of 2.85 to 3.12 min to highlight the relative abundance of DF31 and oxidized species in each sample (Fig. S20†). Similarly, in the biotinylated Sav proteins (absence of Cu), the corresponding mass-shifted ions were not observed after treatment with H_2_O_2_ (Fig. S14 and S15†). Taken together, these data suggest that site-specific oxidation of secondary sphere residues is dependent on the presence of the Cu-cofactor, as well as the proximity and orientation of the Cu center with respect to tyrosine.

### Oxidation of other engineered residues

Based on the structures determined by XRD and the MS results, we sought to test whether the incorporation of a tyrosine residue at another location would lead to increase levels of non-specific protein oxidation and whether the oxidation of tyrosines in the secondary coordination sphere was influenced by their location relative to the Cu cofactor. We therefore incorporated our [Cu^II^-biot-et-dpa] cofactor into a new variant in which K121 was substituted for a tyrosine, producing [Cu^II^-biot-et-dpa⊂2XM-S112A-K121Y-Sav] (3), where Y121 sits 6.7 Å from the copper center as determined by XRD (Fig. S23 and Table S2†). We note that despite being nearly equidistant to the Cu center as in 1, the interplay between tyrosine incorporation and N49 coordination is not observed in 3. This difference is likely because Y121 in 3 is unable to achieve the steric influence exerted by the positioning of Y112 in 1. Upon reacting with peroxide, whole-protein HR-ESI-Q-TOF MS of 3 showed oxidation levels that fell between those observed for 1 and 2 (Fig. 3C). While peaks corresponding to singly and doubly oxidized species increased in relative abundance compared to those same features in 2, the base peak at 16 443.2 *m*/*z* still corresponds to the unoxidized protein, indicating that it remained the dominant species in solution.

The premise that S112Y is modified due to its positioning was supported by LC-MS (and MS/MS) analysis of the trypsin digestion of 2 and 3, where treatment with H_2_O_2_ resulted in only minor modification of the DF31 peptide (Fig. 4F, G and S20†). The surprising result that 3, with a tyrosine at 121, shows less modification of DF31 than 2 suggests that oxidation depends on the orientation and position of the Cu cofactor. We point out that while the oxidation of DF31 is limited in 3, the non-specific oxidation of other residues in 3 is enhanced relative to that in 2 (Fig. 3B and C), an observation that is not yet completely understood. However, comparison of the structures of 1 and 3 reveals differences in their primary coordination spheres, in which the amide residue at N49 no longer coordinates the Cu center in 3.

Based on the observations that positioning of the tyrosine is important for promoting activity, we reasoned that oxidation of phenylalanine residues might also be achievable while maintaining the cofactor geometry and orientation. We were able to successfully prepare and characterize the S112F variant or [Cu^II^-biot-et-dpa⊂2XM-S112F-K121Y-Sav] (4), where the molecular structure determined by X-ray diffraction reveals that F112 can maintain support of the N49 coordination to the Cu cofactor (Fig. S24†). Moreover, the orientation of F112 in 4 is comparable to that of Y112 in 1 (Fig. S25†). However, after several attempts, *in crystallo* reactivity studies of 4 with H_2_O_2_ were inconclusive due to heterogeneity and disorder within the crystals. It is possible that the increased disorder of the F112 structures upon H_2_O_2_ exposure is caused by the lack of a stabilizing H-bond, exhibited by Y112 in 1, with the backbone carbonyl of A121 (Fig. 1A). Nevertheless, both the intact protein mass spectrum (Fig. 3D) and enzymatic digest analysis reveal peaks corresponding to specific oxidation of 4 upon peroxide-treatment (Fig. 3D, 4H and I).

### Reactivity of ArMs with substates

The selective oxidation of S112Y in 1 also indicated that the confinement and positioning of our Cu cofactors within their Sav variants may preclude them from reacting with other substrates. As such, we tested the reactivity of 1, 2 and 3 towards a commonly utilized external substrate, *p*-nitrophenyl-β-d-glucopyranoside (PNPG) (Scheme S2†).^23^ Our biotinylated Cu cofactor alone in solution rapidly reacted with PNPG, while 1, 2 and 3 showed negligible reactivity (Fig. S28†). These results support the idea that sequestration, confinement, and orientation of the cofactor determines its propensity towards reactivity with internal *versus* external substrates similar to what has been found for the Cu cofactor of LPMO and its reactivity.^20,24^

## Conclusions

The selective hydroxylation of sp^2^ and sp^3^ C–H bonds using both mono- and di-nuclear copper cofactors remains an active area of research in both synthetic and biological contexts.^13,25–31^ In this report, we described Cu ArMs with aromatic residues engineered within their secondary coordination spheres and examined their reactivity with H_2_O_2_. Application of XRD methods suggested their ability to selectively oxidize tyrosine residues within the secondary coordination sphere to either a catechol or quinone. Additionally, the rotameric change and coordination of N49 in 1 and 4 also suggested its potential role in reactivity. Note that this type of coordination has not been observed in any other ArMs; however, as mentioned above, a recent structure of pMMO revealed a new potential catalytic site having a similar amide coordination to a single Cu center (Fig. 1B).^5^ It is not yet known if this type of coordination is necessary for function in pMMO, but the lack of coordination in our series of Cu ArMs leads to a decrease in reactivity.

Considering results from our prior work,^18^ it is reasonable to propose that a Cu^II^–OOH species is initially generated upon treatment of our of Cu ArMs with H_2_O_2_. These earlier Cu ArMs with WT Sav allowed us to pinpoint that stabilization of the Cu–OOH unit required a H-bond network that included residues at N49 and S112. These positions are not available for H-bond bonding in 1 and 4 because of mutations at 112 and N49 coordinating to the Cu center. The lack of H-bonds to the Cu^II^–OOH unit produces a more reactivity intermediate that activates the hydroperoxide for oxidation. The fact that we observe site-specific oxidation at 112 suggests that reactivity occurs *via* interaction with an intermediate involving the Cu cofactor which is supported by our H_2_^18^O_2_ labeling studies (Fig. S16†). However, the mechanism(s) followed to produce the oxidized proteins is still uncertain. We probed the reaction using *in situ* electron paramagnetic resonance spectroscopy but these studies did not reveal the accumulation of any organic radical species. We also cannot rule out the production of hydroxyl and/or hydroperoxyl radicals during the reaction and as the source of the observed background non-specific oxidation.

Employing LC-MS methods in combination with enzymatic digestion of our Cu ArMs further supported the selective oxidation of 1 upon the addition of peroxide. Our results demonstrate how the inclusion of aromatic residues within the secondary coordination sphere influences the structure and reactivity of copper ArMs. Moreover, these results may suggest a potential inactivation pathway for LPMOs and other metalloenzymes upon exposure to H_2_O_2_ in the absence of external substrates.^32^ Importantly, oxidation of tyrosine and phenylalanine residues is dependent on its proximity and orientation relative to the artificial metallocofactor. When the location is appropriate, such as in the case of S112Y and S112F, steric constraints shift Cu center towards N49, allowing amide coordination and then S112Y and S112F are readily oxidized upon exposure to H_2_O_2_. In 3, the tyrosine residue at 121, while also proximal to the Cu center, does not exert the same steric interactions to position the Cu center for N49 coordination—and oxidation of Y121 was not observed. These results highlight how subtle changes in the local environment surrounding metallocofactors can influence reactivity.

## Data availability

PDB accession codes: 1: 9CSU, 1 + H_2_O_2_: 9CSV, 2: 9CST, 3: 9CSW and 4: 9E6Z. The data supporting this article have been included as part of the ESI†.

## Author contributions

The concept, experimental studies, and data analysis were performed by K. S. U., A. H. F., and A. S. B. All authors actively participated in manuscript preparation and editing.

## Conflicts of interest

There are no conflicts to declare.

## Supplementary Material

SC-OLF-D4SC06667G-s001
