# Supplementary material for: Selective oxidation of active site aromatic residues in engineered Cu proteins
Source: Chem Sci. 2024 Nov 18;16(1):98–103. doi: 10.1039/d4sc06667g (PMC11587884; doi:10.1039/d4sc06667g)
Supplement: SC-016-D4SC06667G-s001 [file SC-016-D4SC06667G-s001.pdf]

Supplementary Information (SI) for Chemical Science.
This journal is © The Royal Society of Chemistry 2024

Supporting Information

Selective Oxidation of Active Site Aromatic Residues in Engineered Cu Proteins

Kylie S. Uyeda, Alec H. Follmer* and A. S. Borovik*

Department of Chemistry, University of California–Irvine, Irvine, CA 92697-3900

*Corresponding Authors: afollmer@uci.edu, aborovik@uci.edu

Contents

Preparative methods S1
Physical methods S3
Protein crystallography, X-ray diffraction data collection and processing S5
Liquid chromatography mass spectrometry methods S7
Scheme S1. Preparation of [Cu^{II}(biot-et-dpa)] cofactor S11
Scheme S2. Assay for LPMO-like reactivity S11
Figure S1. Molecular models of Cu-Sav proteins used in this study S12
Figure S2. HABA titrations S13
Figure S3. UV-vis Cu-2xm-S112Y + H_2O_2 S14
Figure S4. EPR Cu-2xm-S112Y + H_2O_2 S15
Figure S5. Molecular models of Cu-2xm compared to Cu-2xm-S112Y S16
Figure S6. Atom labeling for indicated bond lengths listed in Table S7 S17
Figure S7. Molecular model of Cu-2xm-S112Y + H_2O_2 with F_o-F_c map S18
Figure S8. Molecular model of Cu-2xm-S112Y + H_2O_2 with two Y112 conformations S19
Figure S9. Chromatograms of intact Cu-Sav variants S20
Figure S10. Mass spectra showing ions of highest abundance of intact Cu-Sav variants S21
Figure S11. Chromatograms of intact apo- and biot- Sav variants S22
Figure S12. Mass spectra showing ions of highest abundance of intact apo- and biot- Sav variants S23
Figure S13. Deconvoluted mass spectra of intact apo- and biot- Sav variants S24
Figure S14. Integrated areas of chromatograms for biot-Sav variants S25
Figure S15. Mass spectra of DF31 for biot-Sav variants S26
Figure S16. Chromatogram, ions of highest abundance and deconvoluted mass spectrum of intact Cu-2xm-S112Y Sav + $^{18}O_2H_2$ S27
Figure S17. Identified *b*/*y* ion fragments for Cu-2xm-S112Y ± H_2O_2 S28
Figure S18. Chromatograms of digested Cu-Sav variants S29
Figure S19. Separately integrated chromatographic peaks containing DF31 and resulting mass spectra S30
Figure S20. Integrated areas of chromatograms for Cu-Sav variants S31
Figure S21. Molecular model of Cu-2xm-S112Y with distances from Cu to other residues in DF31 susceptible to oxidation S32
Figure S22. Molecular model of Cu-2xm-S112Y + H_2O_2 (other residues in DF31 known to be susceptible to oxidation by H_2O_2 with F_o-F_c difference density map) S33
Figure S23. Molecular model of Cu-2xm-S112A-K121Y-Sav S34
Figure S24. Molecular model of Cu-2xm-S112F-Sav S35
Figure S25. Molecular models of Cu-2xm-S112Y comparted to Cu-2xm-S112F S36

Figure S26. Chromatogram, and ion of highest abundance for Cu-2xm-S112F **S37**
Figure S27. Chromatogram and integrated area of chromatogram for Cu-2xm-S112F **S38**
Figure S28. Reactivity with PNPG **S39**
Table S1. EPR simulation parameters of spectra in Figure S3 **S40**
Table S2. X-ray crystallography data processing and refinement statistics **S41**
Table S3. Select bond lengths and angles according to labelling in Figure S5 **S42**
Table S4. Intact protein LC-MS gradient method **S43**
Table S5. Peptide LC-MS gradient method **S43**
Table S6. Possible digest fragments of 2xm-S112Y-Sav **S44**
Table S7. Calculated *m/z* values for *b/y* ion fragments of DF31 **S45**
References **S46**

Preparative Methods

All commercially available reagents were purchased of the highest purity and used as received. Acetonitrile, ethanol, and diethyl ether were degassed with argon and dried by vacuum filtration through activated alumina according to the procedure outlined by Grubbs.[1] Nanopure water was obtained from Millipore Super-Q Plus water purification system, 120 V, 4-stage purification system with pump. Thin-layer chromatography (TLC) was performed on Whatman 250 μm layer 6 Å aluminum-backed silica plates and visualized using ultraviolet (UV) light. Silica gel chromatography was performed using Fisher reagent silica gel 60 (230-400 mesh) with indicated solvents. Biotin pentafluorphenol ester (biot-PFP)[2] and di-(2-picolyl)amine (dpa)[3] were prepared according to literature procedures.

^{1}H and ^{19}F nuclear magnetic resonance (NMR) spectra were recorded at 500 and 400 MHz respectively. ^{1}H NMR spectra are reported in ppm on the δ scale and referenced to tetramethylsilane or solvent residual. The data are presented as follows: chemical shift, multiplicity (s=singlet, d=doublet, t=triplet, q=quartet, quin=quintet, m=multiplet, br=broad), and integration.

Preparation of Cu cofactor. The ligand (biot-et-dpa) was prepared as published previously.[4] ^{1}H (500 mHz, DMSO-d6) δ 8.48 (d, 2H), 7.76 (td, 3H), 7.53 (d, 2H) 7.25 (t, 2H), 6.40 (s, 1H), 6.33 (s, 1H), 4.29 (t, 1H), 4.12 (t, 1H), 3.78 (s, 4H), 3.20 (q, 2H), 3.08 (q, 1 H), 2.81 (dd, 1 H), 2.59 (s, 1H), 2.56 (t, 2H), 2.04 (t, 2H), 1.47 (m, 6H).

The Cu-complex [Cu^{II}(biot-et-dpa)$(NO_3)_2$] was prepared by addition of a solution of $Cu(NO_3)_2$•$3H_2O$ (85.2 mg, 0.353 mmol) in 10 mL ethanol to a solution of biot-et-dpa (150.2 mg, 0.324 mmol) in 50 mL ethanol. The pale blue solution was allowed to stir for 4 hours before concentrating

the solution to ~1 mL followed by precipitation with diethyl ether (**Scheme S1**). Blue solid was collected via filtration, washed with diethyl ether and dried under vacuum.

HR-MS (ES, MeOH) *m/z* calcd for $C_{26}H_{32}CuN_8O_8S$ [M – (NO_3^-)]$^+$ 621.2, found 621.4.

Preparation of Sav variants. The construction of E101Q-K121A-S112Y-Sav (denoted 2xm-S112Y-Sav), E101Q-S112A-K121Y-Sav (denoted 2xm-K121Y-Sav) and E101Q-K121A-S112F-Sav (denoted 2xm-S112F-Sav), was achieved by site-directed mutagenesis (SDM) using the codon optimized E101Q-K121A-pET24a-Sav plasmid, the following primers, and Phusion polymerase.[5]

S_{112}Y_fwd: 5'-CTGACCTATGGCACCACCGAAGCAAATC-3'

S_{112}Y_rev: 5'-GGTGCCATAGGTCAGCAGCCACTGGGTC-3'

S_{112}A_fwd: 5'-CTGACCGCAGGCACCACCGAAGCAAATC-3'

S_{112}A_rev: 5'-GGTGCCTGCGGTCAGCAGCCACTGGGTC-3'

K_{121}Y_fwd: 5'-GCCTGGTATAGCACCCTGGTTGGTCATC-3'

K_{121}Y_rev: 5'-GGTGCTATACCAGGCATTTGCTTCGGAC-3'

SDM reaction mixtures were transformed into *Escherichia coli* Rosetta or bl21 cells followed by rescue with lysogeny broth (LB) media. The solution was spread aseptically onto LB/Kanamycin agar plates and incubated overnight at 37 °C. Single colonies were used to inoculate 10 mL liquid LB/Kanamycin cultures and incubated overnight at 37 °C with shaking at 200 rpm. Of the overnight cultures, 600 μL was saved to prepare glycerol stocks to be stored in 25% glycerol at -80 °C. From the remaining liquid culture, plasmids were isolated using Qiagen Miniprep kit according to manufacturer's instructions. DNA sequencing was performed by Genewiz. Glycerol stocks described above were used to inoculate overnight liquid cultures for expression of Sav mutants. Expression and purification of Sav variants was carried out as reported previously.[6]

Physical Methods

Instrumentation. Electronic absorbance spectra were recorded with an Agilent Cary 50 or Cary 60 UV-vis spectrophotometer in a 1 cm quartz cuvette.

Electron paramagnetic resonance (EPR) spectra were recorded with a Bruker EMX Series EPR spectrometer equipped with an ER4102ST cavity and ER041XG microwave bridge.

HABA titrations. 4'-hydroxyazobenzene-2-carboxylic acid (HABA) is a dye that absorbs light at 506 nm when bound to streptavidin. To determine the average number of biotin binding groups per

protein molecule, a titration of Cu-complex into HABA-bound Sav was performed and displacement of HABA was monitored by the decrease in 506 nm absorbance. To a 1 cm quartz cuvette, 2.4 mL of 8 μM Sav in 200 mM phosphate buffer pH 7 was added. Then 288 μL of a 20 mM (150 equivalents relative to Sav monomer) HABA solution in 200 mM sodium phosphate buffer pH 7 was added and allowed to equilibrate for 5 minutes. A 1 mM aqueous solution of Cu complex was added in 9.6 μL portions until 5 equivalents total were added. Each titration point was spectrophotometrically monitored by following the decrease in absorbance at λ_{max} = 506 nm until no further changes in intensity were observed (**Figure S2)**.

General preparation of Cu-Sav in solution. A solution of lyophilized Sav was prepared in nanopure water. This solution was diluted to 150 μM in MES buffer (50 mM, pH 6), and five equivalents of Cu-complex (750 μM) were added from 10 mM stock solution in nanopure water. Samples were separated from excess Cu complex and concentrated using Amicon Ultra 10K centrifugal filter devices and washed with 5X volume of nanopure water or buffer. The concentration of Cu-Sav stock solution was determined based on ε_M = 167,760 at λ_{max} = 280 nm and diluted to the final working concentration as specifically indicated for each experiment described below.

Electronic absorbance spectroscopy. Cu-Sav was diluted to a final concentration of 150 μM (600 μM Cu) in 50 mM 2-(*N*-morpholino)ethanesulfonic acid (MES) buffer pH 6 in a low-volume 1 cm quartz cuvette. For [Cu^{II}(biot-et-dpa)⊂2XM-S112Y-Sav] (**1**), a feature at λ_{max} = 15675 cm^{-1} (638 nm) was observed, attributed to a Cu d-d transition. This feature was slightly red-shifted in the analogous ArM lacking the tyrosine mutation ([Cu^{II}(biot-et-dpa)⊂2XM-S112Y-Sav], **2**), with λ_{max} = 15480 cm^{-1} (646 nm), indicating a change in the ligand environment of the Cu center. For [Cu^{II}(biot-et-dpa)⊂2XM-K121Y-Sav] (**3**), with a tyrosine mutation in a different location, the Cu d-d transition was observed at λ_{max} = 15723 cm^{-1} (636 nm).

Upon addition of 10 equivalents of hydrogen peroxide (H_2O_2) relative to the Cu concentration to **1,** spectra were collected every 10 minutes (**Figure S3**). Over the first 90 minutes, we observe a hypsochromic shift in the Cu d-d transition as well as the growth of a new shoulder at ~405 nm. The shift in energy of the Cu d-d transition after addition of H_2O_2 over this time suggests a conformational change surrounding the Cu center, which could be important for reactivity. The observation of the

growth of the shoulder at ~405 nm is intriguing as it reminiscent of our previous work suggesting the potential formation of a Cu^{II}-hydroperoxido species.[7]

Electron paramagnetic resonance (EPR) spectroscopy. Cu-Sav was diluted to a final concentration of 250 μM (1 mM Cu) in 50 mM MES buffer pH 6. The solution was allowed to equilibrate for 5 min before addition of 10 equivalents H_2O_2 relative to the Cu concentration. Aliquots of 250 μL were transferred to an EPR tube and flash-frozen in liquid nitrogen. EPR spectra were collected at 77 K, 2.1 mW, 9.4 GHz and averaged over 10 scans (**Figure S4** and **Table S1**). We did not observe changes in the EPR spectra of **1** upon addition of H_2O_2 to suggest formation of a new Cu^{II}–OOH species.

Reactivity with PNPG. To assess reactivity with an external substrate, a previously reported assay for LPMO-like activity was used.[8] Generally, the assay is carried out at pH 10-11 in the presence of 200 equivalents each of *p*-nitrophenyl-β-D-glucopyranoside (PNPG) substrate and H_2O_2 (**Scheme S2**). Cu-Sav was diluted to a final concentration of 100 μM in sodium carbonate buffer (50 mM, pH 10.5). To this solution, 200 equivalents (final concentration 20 mM) PNPG were added and allowed to equilibrate for 5 min. Then 200 equivalents of H_2O_2 (final concentration 20 mM) were added and production of 4-nitrophenolate was monitored by UV-vis at λ_{max} = 405 nm. (**Figure S28**).

Protein Crystallization, X-ray Diffraction Data Collection and Processing

Crystallization of Cu-Sav. Apo-Sav protein was crystallized by the sitting drop vapor diffusion method. Diffraction-quality crystals were grown at ambient temperature and pressure by mixing 2.5 μL protein solution (30 mg/mL for 2xm-S112Y-Sav or 26 mg/mL for 2xm-Sav, 2xm-K121Y-Sav or 2xm-S112F-Sav lyophilized protein in nanopure water) and 2.5 μL crystallization buffer (2.0 M ammonium sulfate, 0.1 M sodium acetate, pH 4). The droplet was equilibrated against a reservoir of 100 μL crystallization buffer solution. Single crystals of Cu-Sav were prepared by soaking apo-Sav crystals soaking buffer (3.0 M ammonium sulfate, 0.1 M sodium acetate, pH 6) for 10 min, followed by a solution of 9 μL soaking buffer mixed with 1 μL of Cu-complex (10 mM Cu-complex, 100 mM $CuCl_2$ in nanopure water) overnight. Upon soaking Sav crystals with Cu-complex, crystals turned from colorless to blue. After soaking in Cu-complex, the blue-colored crystals were transferred to soaking buffer for 10 min to remove excess Cu-complex and Cu salts, then to a solution of 9 μL soaking buffer

mixed with 1 μL H_2O_2 (10 mM) for 5–10 min. Crystals were cryoprotected in Paratone oil (Hampton Research) and shock frozen in liquid nitrogen.

X-ray diffraction data collection and processing. X-ray diffraction data were collected at the Advanced Light Source (BL 5.0.3, 5.0.2 and 5.0.1) or the Stanford Synchrotron Radiation Lightsource (SSRL, BL 12-1 or 12-2). Diffraction images were integrated with iMosflm[9] (**1**, **2**, **3**, **1**+H_2O_2) or XDS[10] (**4**) and scaled with AIMLESS (ccp4 Suite).[11] For molecular replacement, Phaser-MR (PHENIX Suite)[12] was used with PDB: 2QCB with water molecules removed as the input model.

Structural refinement was carried out in PHENIX.REFINE[12,13] and COOT.[14] Restraints for biot-et-dpa (ligand ID: QG7) were created using eLBOW (PHENIX Suite). For water and solvent picking, electron density, and structure visualization, the software COOT[14] was used. Figures were drawn with PyMOL (Schrodinger, LLC Version 2.5.2) Crystallographic details, processing, and refinement statistics are given in **Table S2.**

Overall structures. Apo-crystals of Sav soaked with Cu-complex constituted space group $I4_122$ with unit cell parameters reported in **Table S2**. One Sav monomer was obtained per asymmetric unit after molecular replacement. Protein residues 2–11 and 135–159 of the N- and C- termini, respectively, were not resolved in electron density, presumably due to disorder.

General Cu-complex modelling. For all structures of apo-Sav crystals soaked with Cu-complex the following general observations were made: i) residual electron density in the F_o-F_c map in the biotin-binding pocket, and ii) one (for **1**, **3** or **4**) or two (for **2**) peaks in an anomalous density map in the biotin vestibule superimposed with the electron density peak. The residual electron density was fit with the Cu-complex which projected Cu to the position of the strong anomalous density peak. In **1**, the occupancy of the Cu center was refined to 0.65, and 0.74 with H_2O_2. In **3**, the occupancy of the Cu center was refined to 0.63. In **4**, the occupancy of the Cu center was refined to 0.78. In **2**, the occupancy of the two copper centers were refined to 0.66 and 0.3. Due to overlap of two cofactor conformations and the uncertainty regarding the geometry of the second conformation coupled to its low occupancy, the deposited structure only contains the model of the major Cu-cofactor conformation (**Figure S5A**). In **1**, we also observed residual positive electron density in the F_o-F_c maps surrounding the Cu center. This density was fit to an acetate molecule coordinated to the Cu center in a κ^1 fashion and a water molecule within H-bonding proximity to the acetate molecule. With H_2O_2, this site is occupied by a water molecule. Similarly, in **4**, the residual positive electron density in the F_o-F_c map was fit to an acetate molecule coordinated to the Cu center in a κ^1 fashion.

Modeling N49. For structures of **2** and **3**, residue N49 is modeled as one rotamer. This rotamer is in the same conformation as in our previously reported structures of $[Cu^{II}$(biot-et-dpea)$H_2O)_2]^{2+}$⊂WT-Sav or $[Fe^{III}$(biot-et-dpa)$(OH_2)_2(\kappa^1\text{-}O_{E112})$⊂2xm-S112E-Sav, where the amide likely participates in H-bonding with R84.[4,7] For all structures of **1**, we first modeled N49 in this conformation, but observed negative difference density in the F_o-F_c map surrounding the modelled amide as well as nearby residual positive difference density in the F_o-F_c map suggesting a second conformation of N49. The occupancy of this second N49 conformation was refined to 0.61, consistent with the refined Cu occupancy (0.65). The distance from the Cu center to the heteroatom is 2.4 Å. Due to the inability of the X-ray diffraction experiments to unambiguously determine N vs O coordination, we modeled this as a Cu-κ^1-O_{N49} based on the Cu-heteroatom bond distance as well as previously published reports of the Cu_D site in pMMO[15] and stellacyanin[16] active site structures. With H_2O_2 addition, the occupancy drops to 0.51, about two-thirds of the refined Cu occupancy (0.74). Similarly in **4,** N49 was modeled in two conformations, where the occupancy of the bound conformation was refined to 0.58. Of all Cu-Sav variants reported by our group to date, **1** and **4** with F or Y introduced at position 112 are the only ArMs that exhibits N49 in this second conformation, and the N49 coordination is disrupted to some extent by H_2O_2 addition.

Modeling S112Y. The first 90 degrees in phi rotation for further processing to mitigate possible effects of radiation damage. In this case, the positive density in the F_o-F_c map surrounding S112Y is only observed in **1** with H_2O_2 addition (**Figure S7**). This was best modeled as S112Y covalently modified at the *meta* position. Given the proximity and orientation to the backbone carbonyl of A121 suggesting H-bonding interactions (shown in black dashed lines in **Figure 2**), the oxidized Y112 may be a catechol or semiquinone species. However, we also modeled S112Y in two rotamer positions of equal occupancies with no meaningful change in crystallographic refinement statistics (**Figure S8** and **Table S3**). The deposited structure represents a model of the oxidized Y112 corroborated by the LC-MS and trypsin digestion experiments described below.

Adventitious Cu binding. In **2**, we observe two additional peaks in the anomalous difference map 2.64 Å from $N\eta_{H87}$ and 1.9 Å from $N\eta_{H127}$ which were modeled as Cu atoms. The occupancies were refined to 0.94 and 0.20, respectively. In **3**, we observe one additional peak in the anomalous difference map 2.47 Å from $N\eta_{H87}$ which was modeled and refined as a Cu atom with 0.71 occupancy.

Liquid Chromatography/Mass Spectrometry (LC/MS) Methods

Instrumentation. Mass spectra of synthesized small-molecule intermediates were measured with an ACQUITY UPLC H-class system, single quadrupole QDa (Waters Corporation) or orthogonal acceleration time-of-flight (oa-TOF) LCT Premier (Waters Corporation). Mass spectra of intact proteins or peptides were measured with an ACQUITY ultra-performance liquid chromatography (UPLC) H-class system, Xevo G2-XS quadrupole time-of-flight (QTOF) (Waters Corporation).

Sample preparation for mass spectral studies. Cu-Sav was diluted to a final concentration of 150 μM in 50 mM MES buffer pH 6. Hydrogen peroxide (100 mM) was added to final concentration of 6 mM (10 equivalents relative to Cu-complex). Samples were allowed to incubate with H_2O_2 for 1 hour at room temperature.

Intact Cu-Sav was separated from remaining H_2O_2 and buffer salts in reaction mixtures using Amicon Ultra 10K centrifugal filter devices and washed with 3X volume of nanopure water. The resulting Cu-Sav solution was boiled at 100 °C for 15 min to dissociate Cu-complex. Intact apo-Sav was separated from dissociated Cu-complex using Amicon Ultra 10K centrifugal filter devices and washed with 3X volume of nanopure water. Samples were diluted in nanopure water for LCMS.

Isotope labeled H_2O_2 ($^{18}O_2H_2$, 90% isotope labeled, 2-2.4% solution in DI water) was obtained from Icon Isotopes. Cu-2xm-S112Y-Sav prepared as described above, incubated with $^{18}O_2H_2$ (6 mM, assuming 2% solution in water), and prepared for LCMS as described above.

Analysis of protein modification. Intact apo-Sav sample was analyzed by LC-MS. Proteins were separated away from reaction buffer salts using a phenyl guard column at 45 °C (ACQUITY UPLC BEH Phenyl VanGuard Pre-column, 130 Å, 1.7 μm, 2.1 x 5 mm, Waters Corporation). The 5 min LC method used flow rate of 0.2 mL/min of a gradient of 0.1% formic acid in water (Buffer A), and acetonitrile (Buffer B) according to gradient in **Table S4**.

The Xevo Z-spray ionization source was operated in positive MS resolution mode, 500–4000 Da, with a capillary voltage of 3000 V and cone voltage of 40 V (NaCsI calibration, Leu-enkephalin lock-mass). Nitrogen was used as the desolvation gas at 350 °C and total flow of 800 L/h. Retention times are given in minutes. The major peak of the chromatogram was selected for integration for further analysis (**Figures S9** and **S11**). Total average mass spectra were reconstructed from the charge state ion series using the MaxEnt1 algorithm from Waters MassLynx software V4.1 SCN949 according to

manufacturer's instructions.[17] The ions of highest abundance from this charge state ion series are shown in **Figures S10 and S12**.

Enzymatic digestion and LC-MS/MS analysis. Apo-Sav prepared as described above was subjected to solution digestion with trypsin gold (Promega) at 37 °C in ammonium acetate pH 7.8 for 8 hrs, following manufacturer's recommendations. All LC-MS/MS experiments were performed using the same Waters Xevo Q-TOF instrumentation and buffer system as the intact protein analysis listed above. Separation of peptides was performed by reverse-phase chromatography at 45 °C using a reverse-phase C4 column (ACQUITY UPLC Protein BEH C4 column, 300 Å, 1.7 μm, 2.1 x 50 mm, Waters Corporation) and the solvent gradient listed in **Table S5**. A 0.5 min waste-divert method was implemented to remove buffer salts.

The Xevo Z-spray source was operated with a capillary voltage of 3000 V and a cone voltage of 40 V (NaCsI calibration, Leu-enkephalin lock-mass). Nitrogen was used as the desolvation gas at 350 °C and a total flow of 800 L/h. Data was acquired across the 100–2000 Da range in MSe continuum mode with alternating 0.5 sec scans at alternating collision energy: low energy, 0 V, and a high collision energy ramp (15–45 V). The high energy data corresponds to the MS/MS secondary fragmentation of the low energies scans of a similar time.

The data was processed for exact mass peptide matching and b/y ion fragmentation confirmation using BioPharmaLynx software (Version 1.3.5, Waters Corporation). The software generates *in silico* peptide digestion data including all potential modifications and maps the real data with a 30-ppm error tolerance to the theoretically calculated masses. From **1**, BioPharmaLynx was used to identify the modified fragment of highest abundance compared to control incubated without H_2O_2 addition. Of the 44 possible peptide fragments resulting from trypsin digestion, DF31 was identified as an oxidized fragment. From DF31, $[DF31]^{3+}$ and $[DF31]^{2+}$ were identified with theoretical m/z 1080.6 and 1620.8, respectively, which elute at 3.07 min (**Table S6**). In samples of **1** treated with H_2O_2, we observe $[DF31+16]^{3+}$ with theoretical m/z 1086.5, eluting at 2.87 min, and $[DF31+32]^{3+}$ with theoretical m/z 1091.2, eluting at 2.97 min (**Figures S17–19**), supporting the single and double oxidation of DF31. The chromatogram was integrated over this entire range, from 2.85 to 3.12 min, to capture the relative abundance of both the unoxidized and oxidized DF31 species for further processing **(Figure 4).** The same areas of the chromatograms were integrated for further processing, to highlight the relative of DF31 and oxidized DF31 in each sample (**Figure S19**).

DF31 contains residues 104–132, supporting the notion that Y112 may be oxidized; however, this DF also contains other residues known to be susceptible to oxidation by H_2O_2 (H127, W108, and

W120). MS/MS secondary fragmentation data were further analyzed for b/y ion fragment identification, and fragments b8, b7, $[y20 +16]^{2+}$, $[y21 +16]^{2+}$, and $[y22 +16]^{2+}$ were identified, confirming oxidation of residue Y112 (**Figure S17** and **Table S7**). This analysis of b/y ion fragmentation also indicates oxidation of W120 to some extent, and oxidation of H127 could not be ruled out.

Although we could not verify the specific oxidation of *only* Y112 in **1**, comparison to biot-2xm-S112Y-Sav (no copper) supports the idea that the Cu-cofactor enhances oxidation of DF31 upon addition of H_2O_2. In biot-2xm-S112Y-Sav, the oxidized fragments $[DF31+16]^{3+}$, $[DF31+32]^{3+}$, $[DF31+16]^{2+}$ and $[DF31+32]^{2+}$ could not be identified, suggesting that DF31 is not oxidized by H_2O_2 in the absence of Cu. Unoxidized fragments $[DF31]^{3+}$ and $[DF31]^{2+}$ could be identified with theoretical *m/z* 1080.6 and 1620.8, respectively, eluting at 3.1 min.

From **2**, $[DF31]^{3+}$ was identified with 1055.5 *m/z*, which elutes at 3.1 min. After incubation with H_2O_2, a small amount of $[DF31+16]^{3+}$ and $[DF31+32]^{3+}$ was also observed, though their relative abundance compared to unoxidized species is significantly less than in the oxidized **1** variant (**Figure 4**). From **3**, $[DF31]^{3+}$ was identified with 1081.2 *m/z*, which elutes at 3.06 min. The corresponding mass-shifted ions $[DF31 + 16]^{3+}$ and $[DF31 + 16]^{2+}$ were not observed in these samples treated with H_2O_2, suggesting that DF31 is not oxidized in **3** (**Figure 4**).

Similarly, in all biot-Sav variants studied, the corresponding mass-shifted ions $[DF31 + 16]^{3+}$ and $[DF31 + 16]^{2+}$ were not observed after treatment with H_2O_2 (**Figures S14** and **S15**). It is interesting to note that overall oxidation of intact Sav is enhanced in all apo-Sav variants compared to biot-Sav variants, which is possibly due to ligand binding precluding access of H_2O_2 to oxidizable residues (**Figure S13**).

Treatment of **1** with $^{18}O_2H_2$ resulted in the growth of two peaks in the mass spectrum at +17.5 *m/z* and +35.1 *m/z*, corresponding to the incorporation of on one or two ^{18}O atoms into monomeric Sav derived from 90% isotope labeled $^{18}O_2H_2$. (**Figure S16**). After trypsin digestion, $[DF31]^{3+}$, $[DF31+16]^{3+}$ and $[DF31+32]^{3+}$ were identified; however, we could not deconvolve oxidized fragments containing ^{18}O versus ^{16}O.

Taken together, these data suggest that site-specific oxidation of secondary sphere amino acids is dependent on 1) the presence of the Cu cofactor, and 2) the proximity and orientation of the Cu center with respect to Tyr.

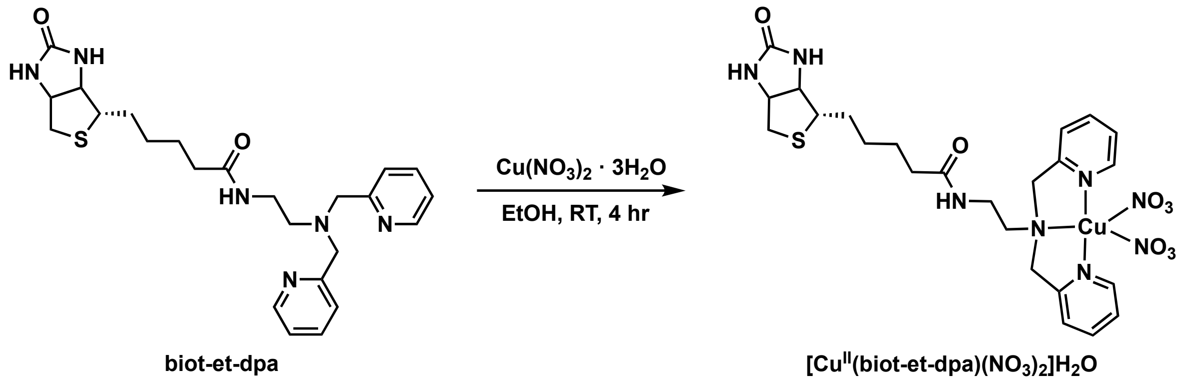

Scheme S1. Preparation of Cu cofactor

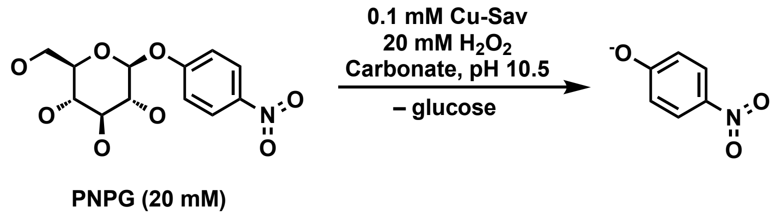

Scheme S2. Assay for LPMO-like reactivity with external substrate *p*-nitrophenyl-β-D-glucopyranoside (PNPG) to produce 4-nitrophenolate (UV-vis λ_{max}=406 nm)

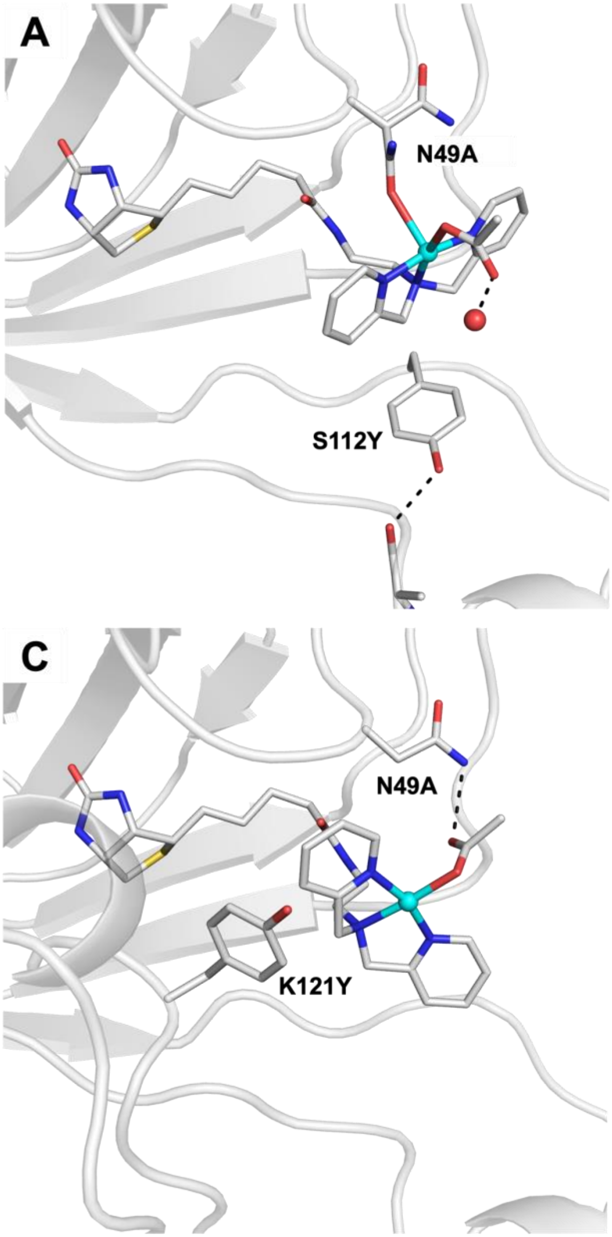

Figure S1. Molecular models of (A) **1** (PDB: 9CSU), (B) **2** (PDB: 9CST), (C) **3** (PDB: 9CSW), and (D) **4** (PDB: 9E6Z).

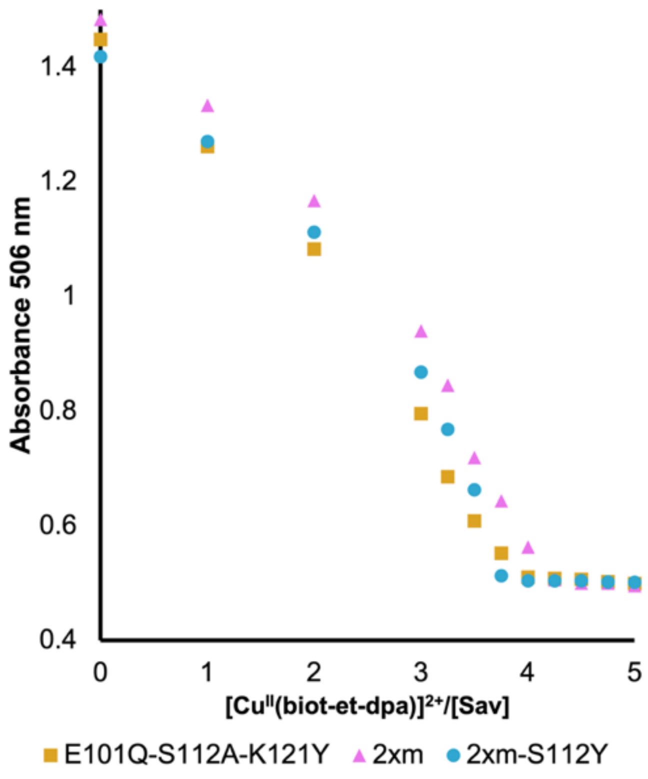

Figure S2. HABA titrations of Cu-et-dpa into Sav variants confirming an average of ~4 biotin-binding sites per Sav molecule for each of the Sav variants used in this study.

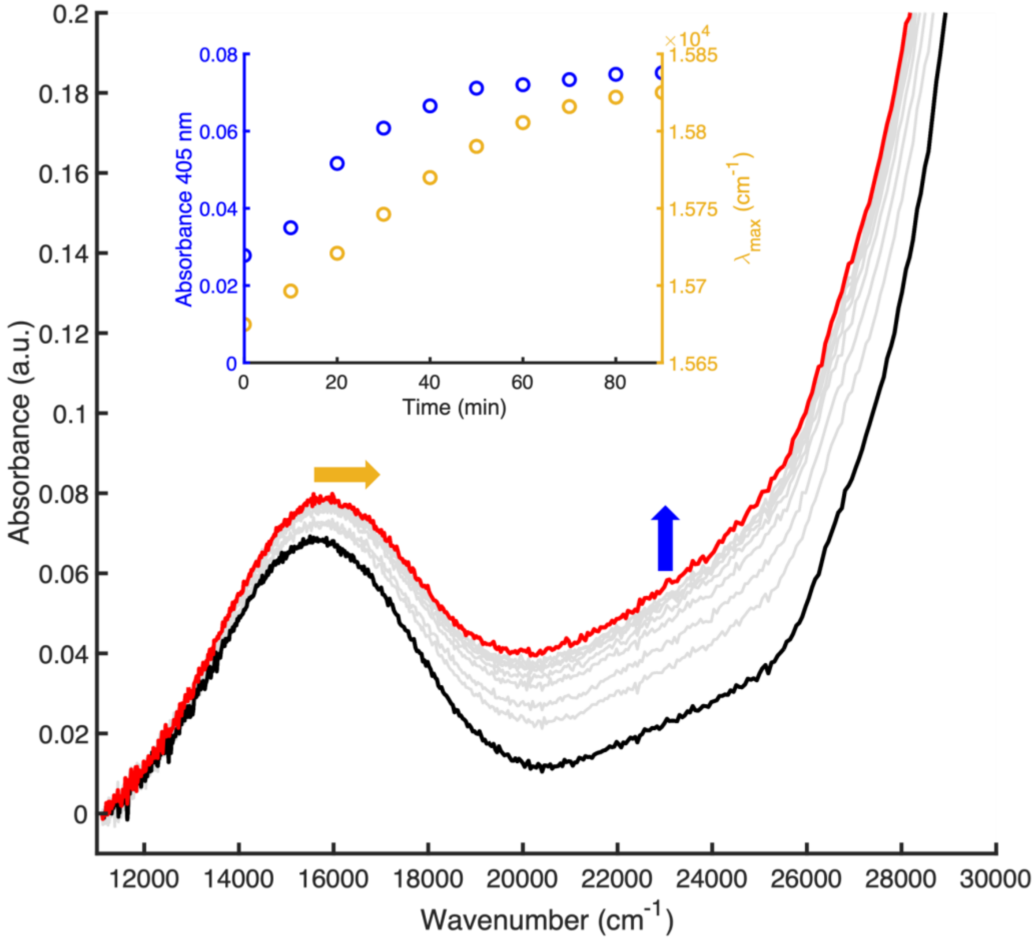

Figure S3. UV-vis spectra of **1** (black), **1** + 10 eq H_2O_2 every 10 min (gray) and after 90 min (red). The shoulder at 24691 cm^{-1} (~405 nm) grows in over time (inset, shown in blue) and the Cu d-d band shifts from λ_{max} = 15675 cm^{-1} to 15825 cm^{-1} (inset, shown in yellow).

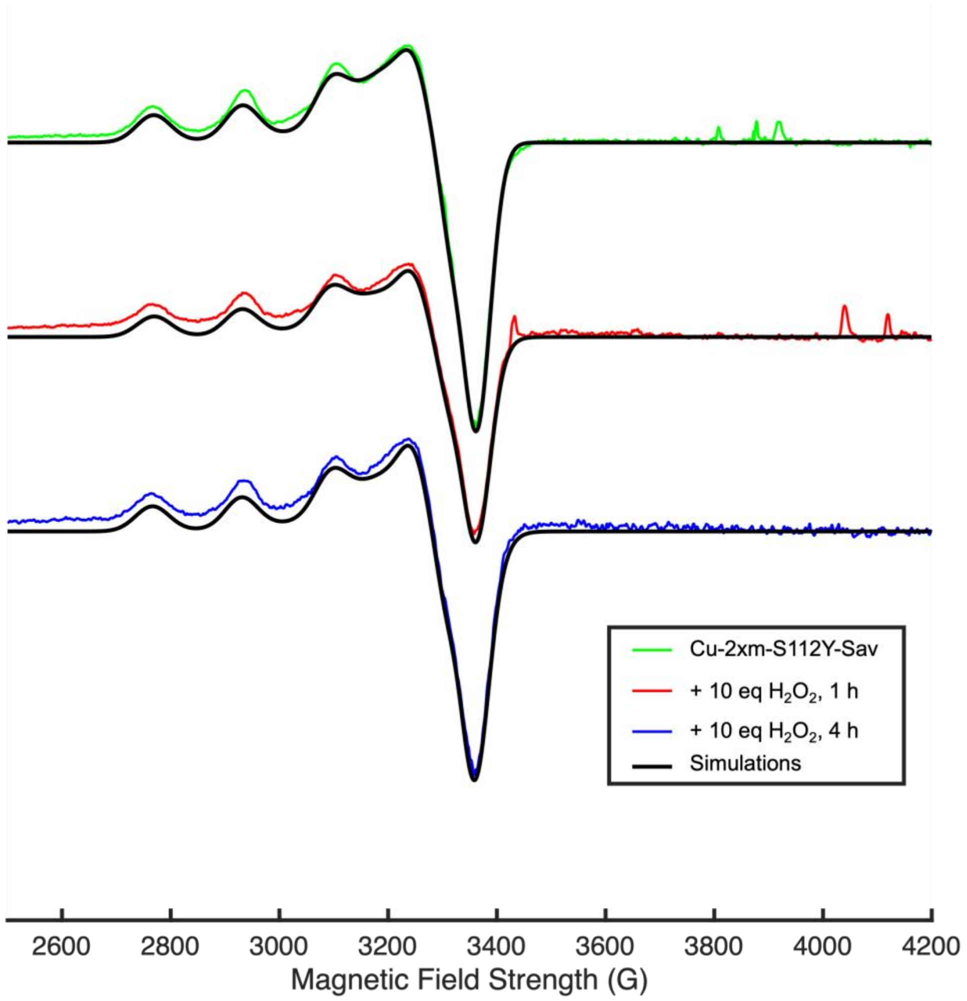

Figure S4. X-band EPR spectra of **1** (green), with 10 eq H_2O_2 after 1 h (red) and after 4 h (blue). Simulations are in black lines. The *g*-values and A-values for simulations are given in Table S1.

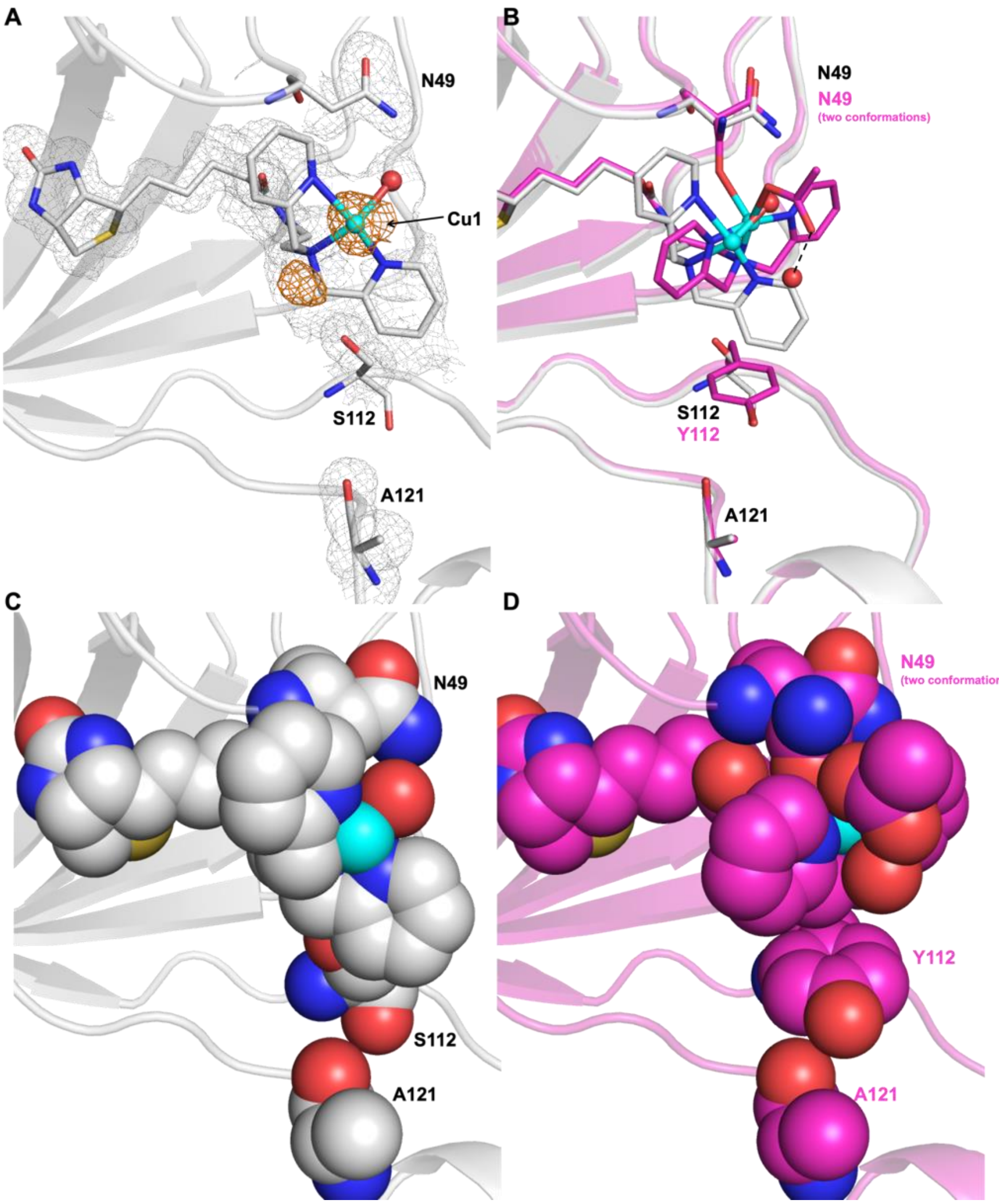

Figure S5. (A) Molecular model of **2** (PDB: 9CST). The protein is displayed as a cartoon and the Cu complex as well as residues N49, S112, and A121 as sticks. Copper is colored in cyan and water molecules are colored as red spheres. The position of the Cu complex as well as residues N49, S112 and A121 are indicated by the $2F_o$–F_c electron density shown in grey mesh contoured at 1σ, and the Cu positions are indicated by the anomalous electron density shown in orange mesh contoured at 3σ. Only the major conformation of the Cu complex is modeled at 66% occupancy.

(B) Overlay of molecular structures of **2** (carbon atoms represented in grey) and **1** (carbon atoms represented in pink, PDB: 9CSU). The protein is displayed as a cartoon and the Cu complex as well as N49, S/Y112, and A121 as sticks. Copper is colored in cyan and water molecules are colored as red spheres. Hydrogen bonds suggested in black dashed lines.

Sphere packing models of (C) **2** and (D) **1** suggest incorporation tyrosine at position 112 induces a preferential orientation on the cofactor, as well as shift in the copper position by 0.6 Å towards N49 compared to Cu-2xm-Sav.

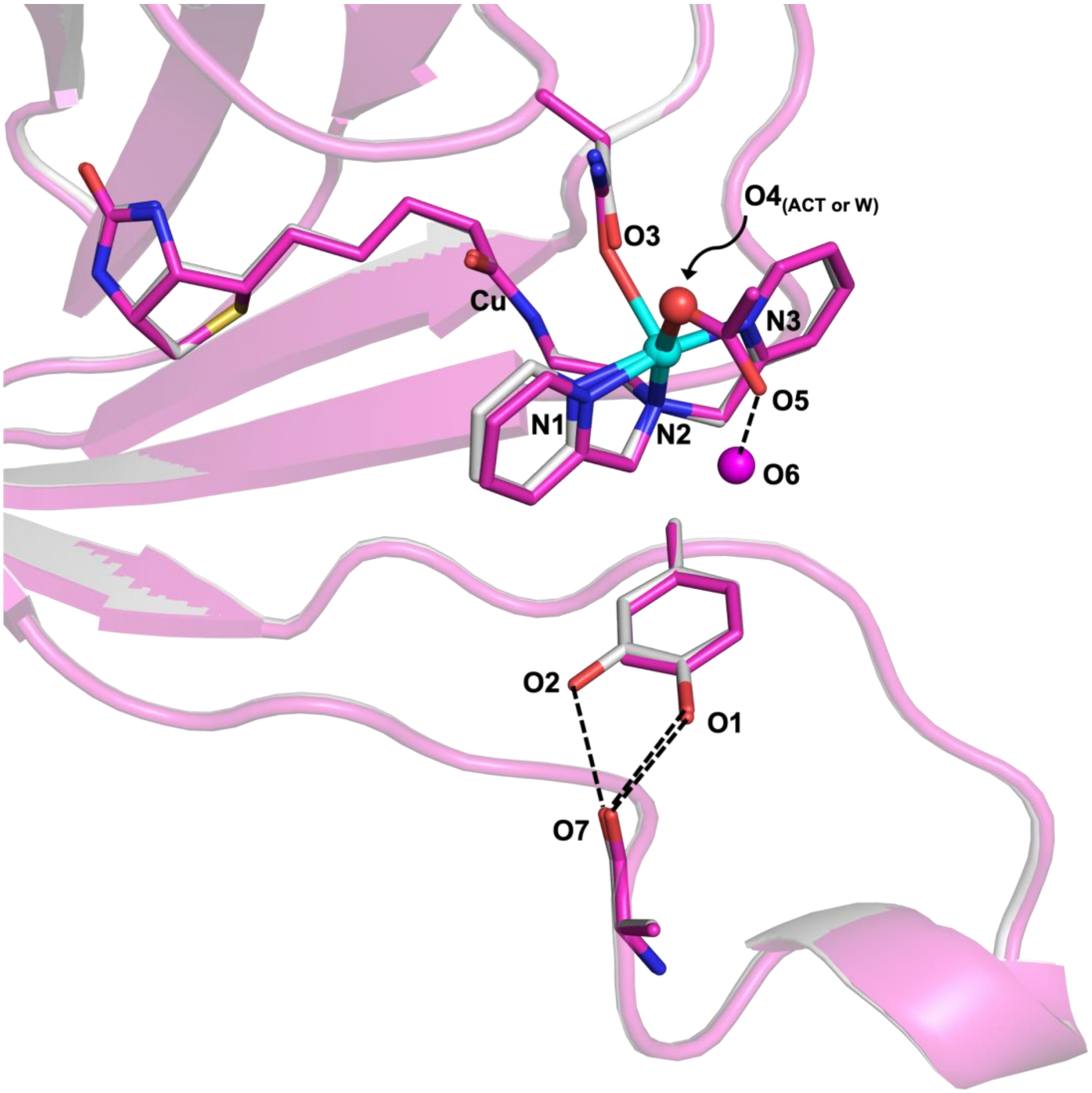

Figure S6. Molecular model of **1** (pink, PDB: 9CSU) and **1** + H_2O_2 (grey, PDB: 9CSV). Atom labeling to indicate select bond lengths and angles listed in Table S7.

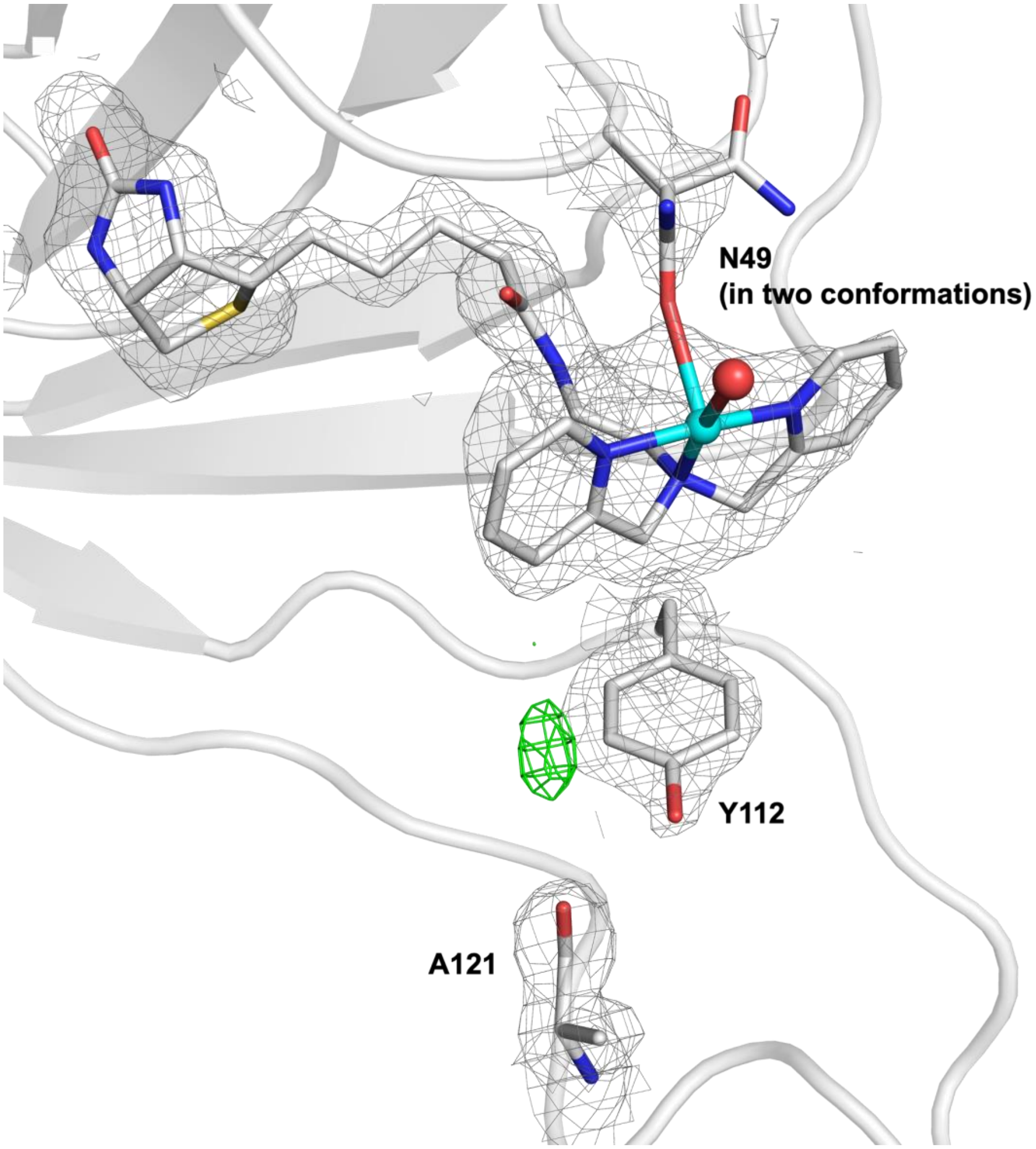

Figure S7. Molecular structure of **1**. The protein is displayed as cartoon representation and the Cu complex as well as residues Y112 and A121 as sticks. Copper is colored in cyan and water molecules are colored in red spheres. Positive difference density in the F_o–F_c map around Y112 contoured at 3σ shown in green mesh. Negative difference density in the F_o–F_c map around Y112 contoured at 3σ shown in red mesh. The position of the Cu complex as well as residues Y112 and A121 are indicated by the $2F_o$–F_c electron density shown in grey mesh contoured at 1σ.

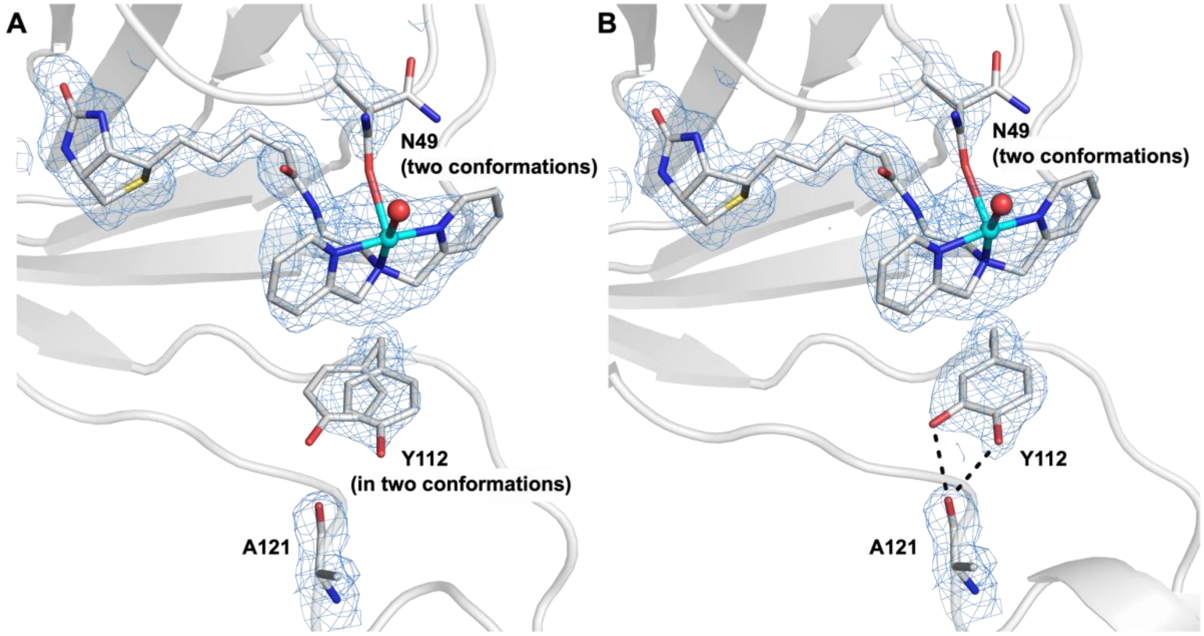

Figure S8. (A) Molecular model of **1** + H_2O_2 with Y112 modeled in two rotamer conformations (not deposited to PDB), and (B) molecular model of **1** + H_2O_2 with Y112 modeled as a catechol-like species (Same as Figure 2B, PDB: 9CSV). The protein is displayed as a cartoon and the Cu complex as well as N49, Y112 and A121 as sticks. Copper is colored in cyan and water molecules are colored as red spheres with the $2F_o$–F_c electron density shown in blue mesh contoured at 1σ. Hydrogen bonds are suggested in black dashed lines. Refinement statistics for this model in comparison to the model of oxidized Y112 are listed in Table S6.

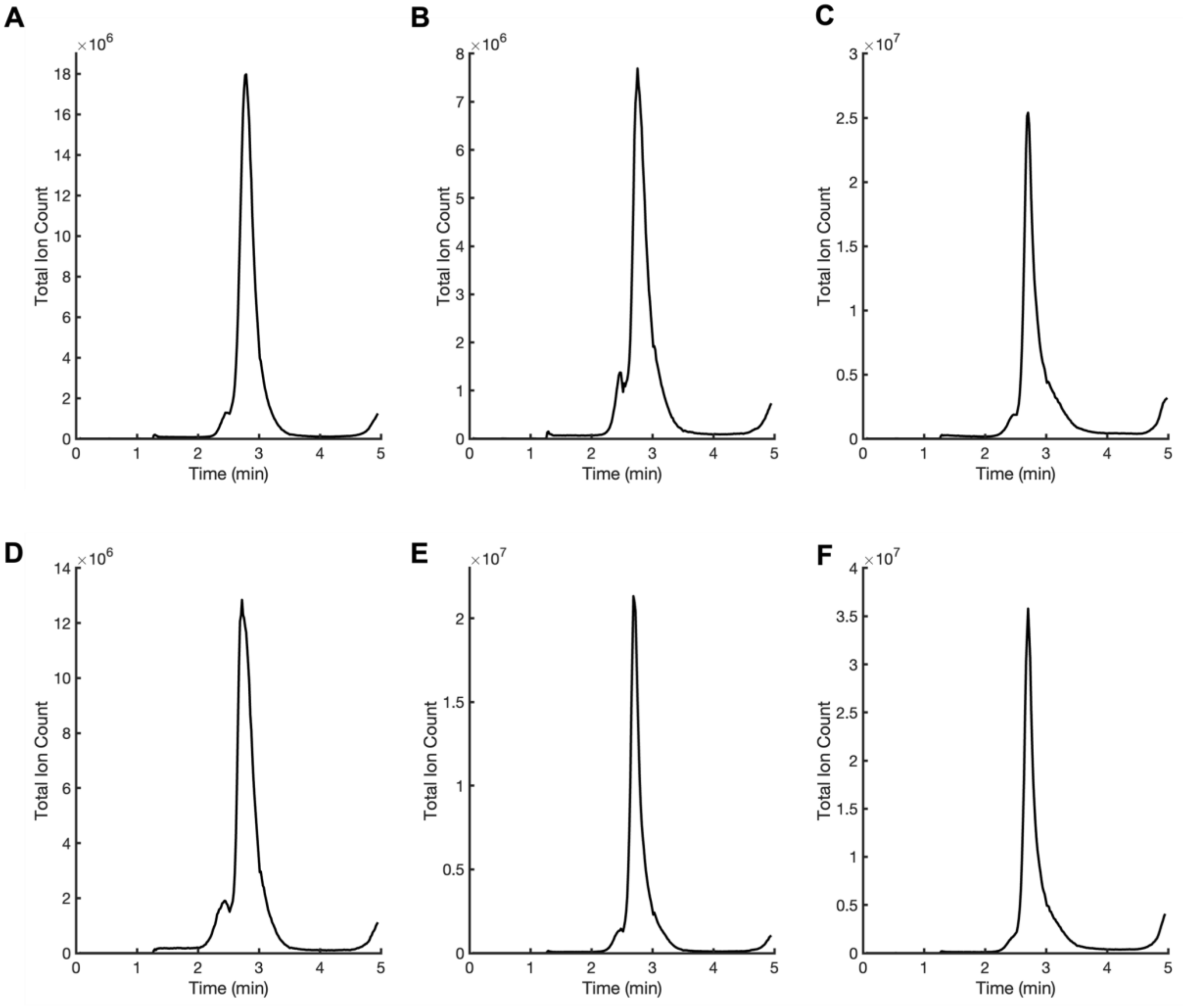

Figure S9. Chromatograms of (A) **2**, (B) **1**, (C) **3**, (D) **2** + H_2O_2, (E) **1** + H_2O_2, and (F) **3** + H_2O_2. The major peak of each chromatogram was integrated at full width/half maximum for further analysis.

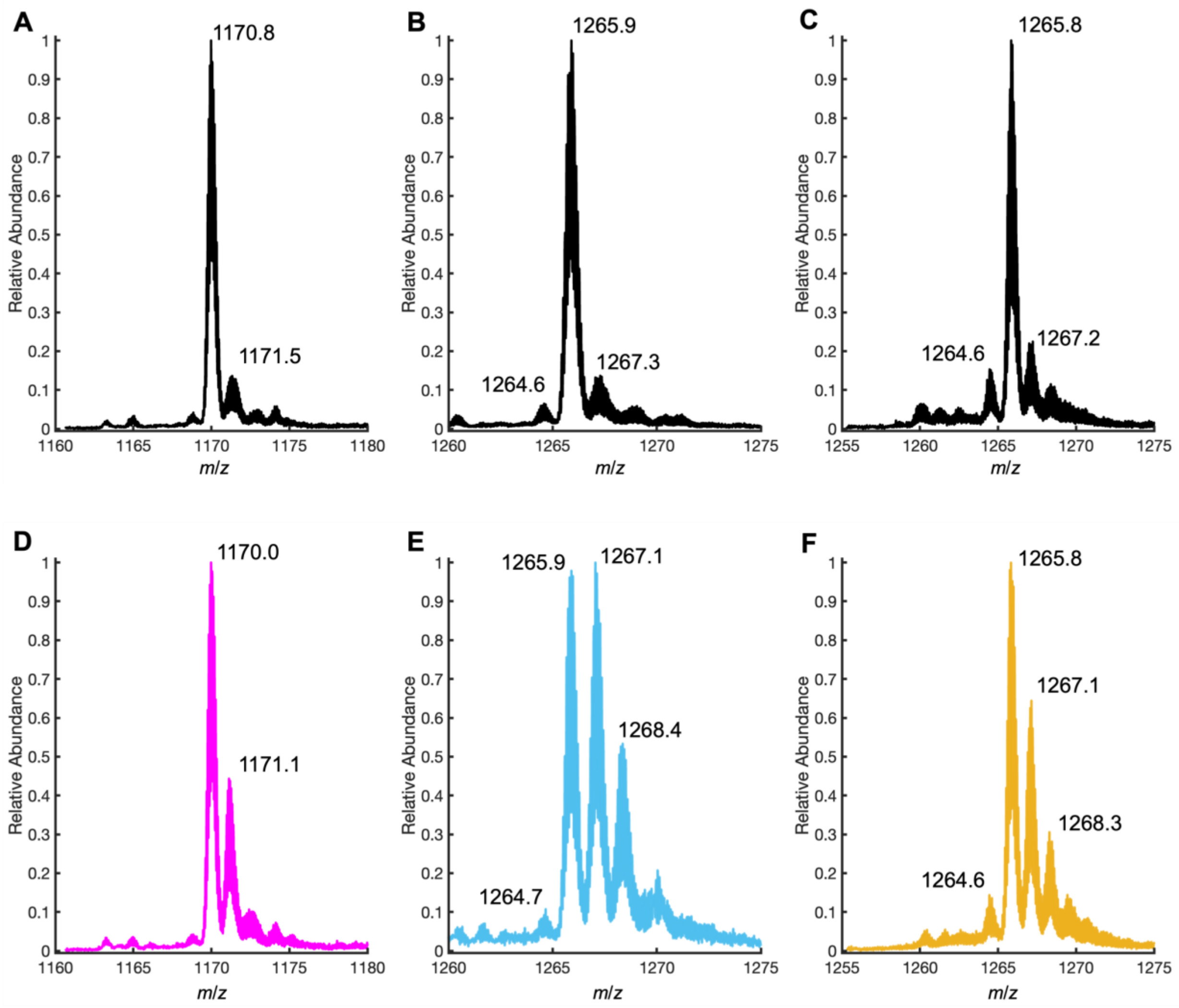

Figure S10. ESI-Q-TOF positive ion mode mass spectra showing ions of highest abundance from integration of the major peaks of the chromatograms of (A) **2**, (B) **1**, (C) **3**, (D) **2** + H_2O_2, (E) Cu-**1** + H_2O_2, and (F) **3** + H_2O_2. The charge-state ion series containing these ions were used to reconstruct total average mass spectra shown in Figure 3.

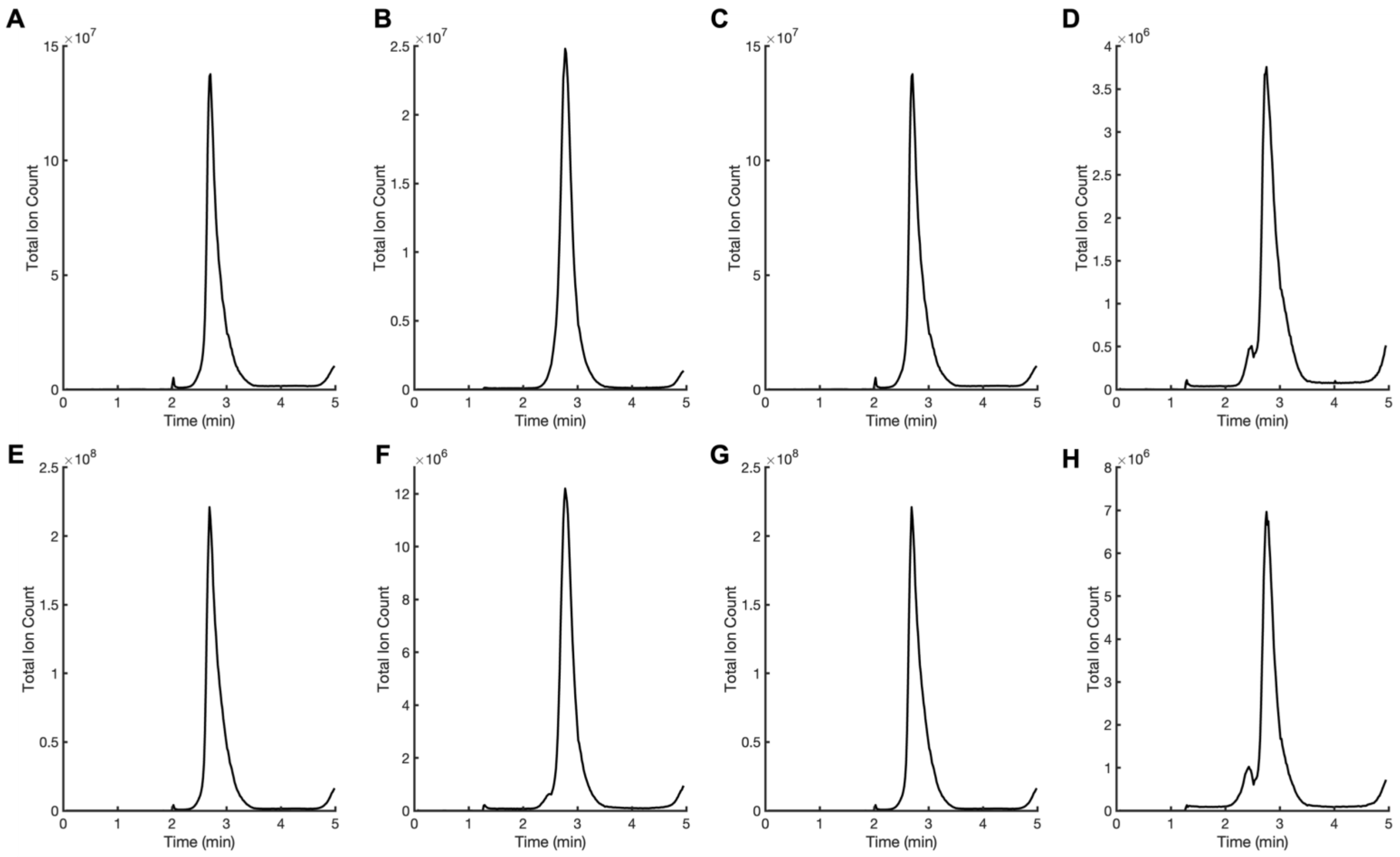

Figure S11. Chromatograms of (A) apo-2xm-Sav, (B) biot-2xm-Sav, (C) apo-2xm-S112Y-Sav, (D) biot-2xm-S112Y-Sav, (E) apo-2xm-Sav + H_2O_2 , (F) biot-2xm-Sav + H_2O_2, (G) apo-2xm-S112Y-Sav + H_2O_2, and (H) biot-2xm-S112Y-Sav + H_2O_2. The major peak of each chromatogram was integrated at full width/half maximum for further analysis.

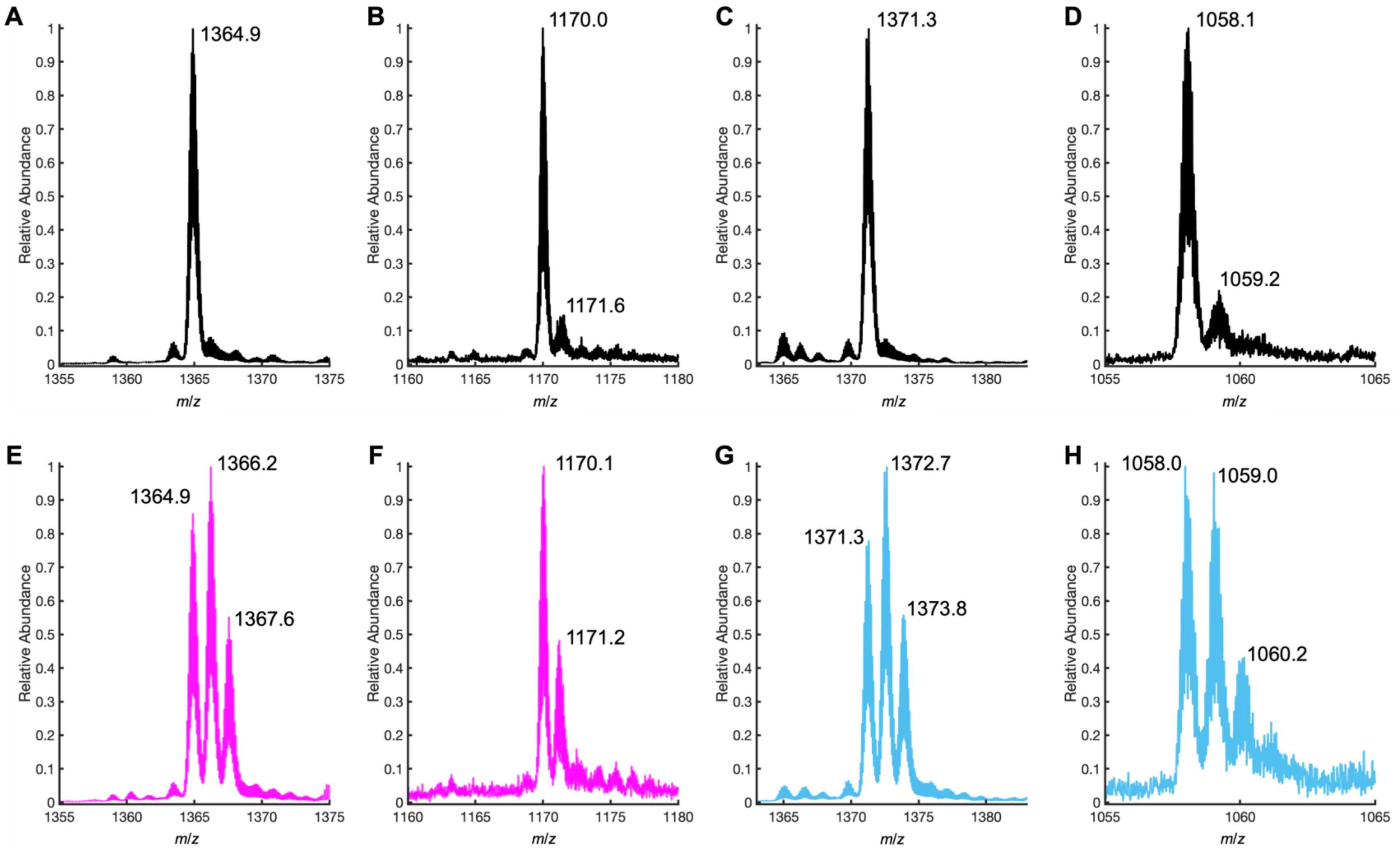

Figure S12. ESI-Q-TOF positive ion mode mass spectra showing ions of highest abundance from integration of major peak of chromatogram of (A) apo-2xm-Sav, (B) biot-2xm-Sav, (C) apo-2xm-S112Y-Sav, (D) biot-2xm-S112Y-Sav, (E) apo-2xm-Sav + H_2O_2, (F) biot-2xm-Sav + H_2O_2, (G) apo-2xm-S112Y-Sav + H_2O_2, and (H) biot-2xm-S112Y-Sav + H_2O_2. The charge-state ion series containing these ions were used to reconstruct total average mass spectra shown in Figure S12.

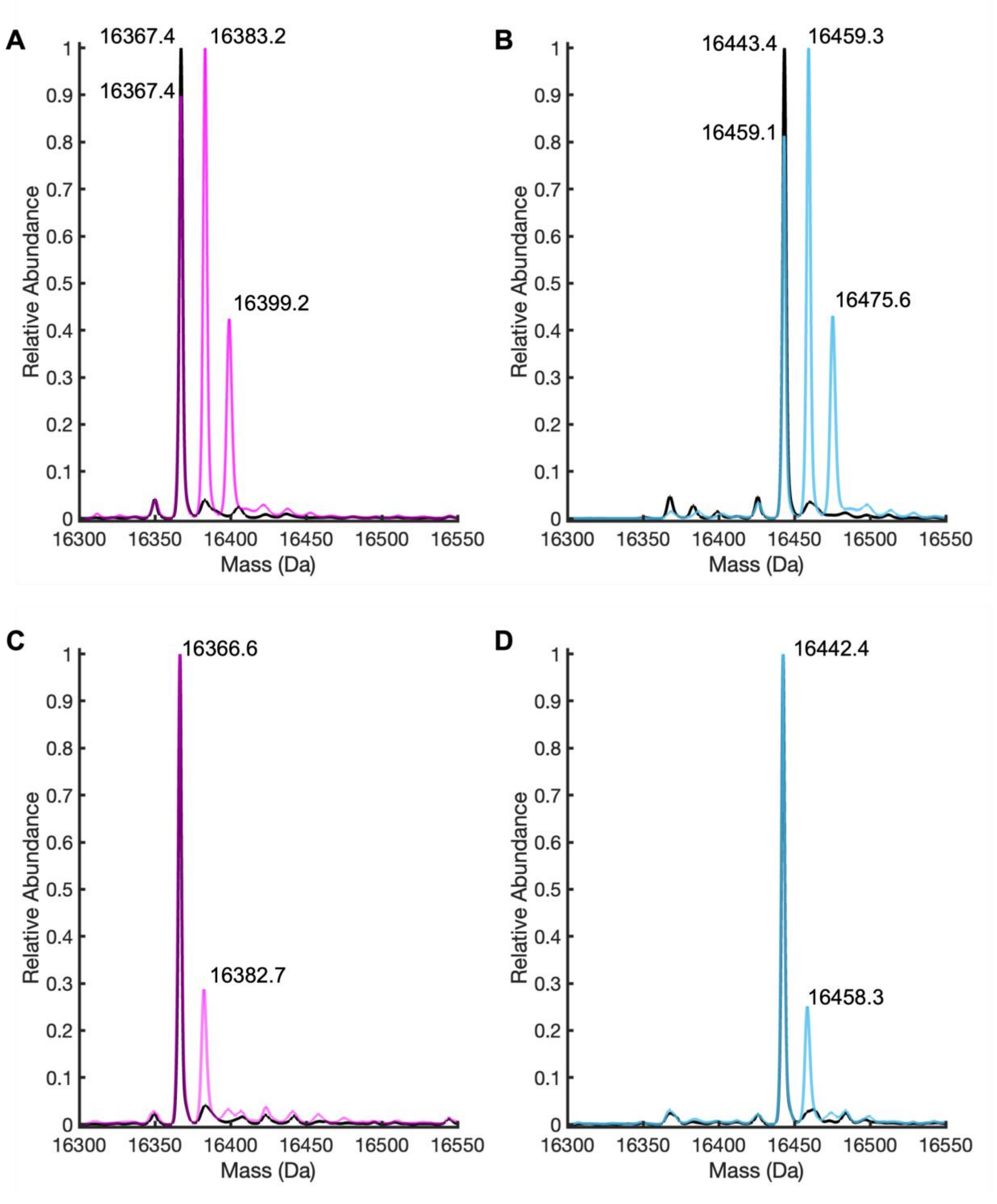

Figure S13. Deconvoluted ESI-Q-TOF positive ion mode mass spectra of (A) apo-2xm-Sav, (B) apo-2xm-S112Y-Sav, (C) biot-2xm-Sav, and (D) biot-2xm-S112Y-Sav. Before addition of H_2O_2 shown in black and after 1 h incubation with 10 eq H_2O_2 shown in pink/blue for each panel. Overall oxidation of intact Sav is enhanced in all apo-Sav variants compared to biot-Sav variants, which is possibly due to ligand binding precluding access of H_2O_2 to oxidizable residues

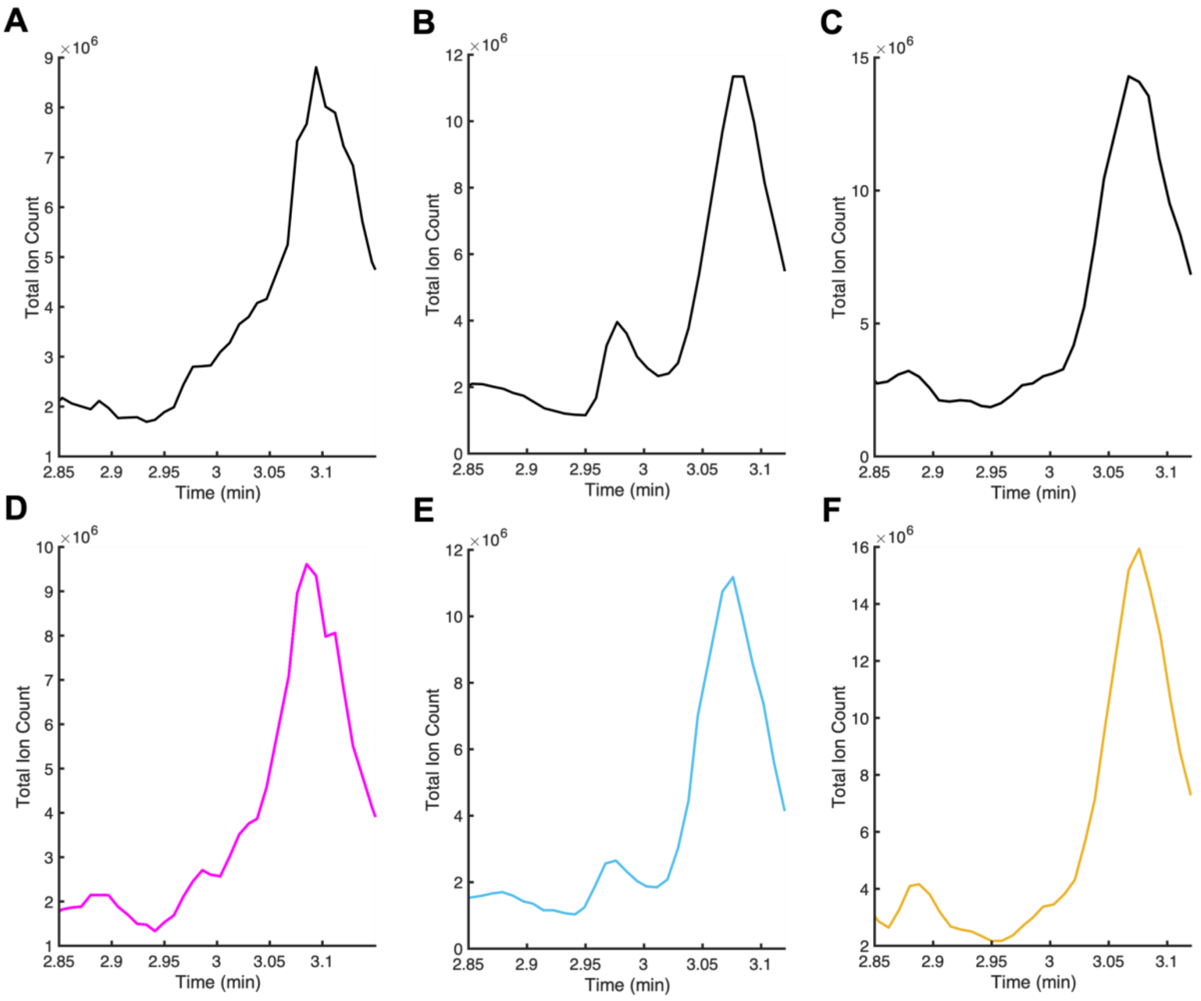

Figure S14. Area of chromatogram after trypsin digestion of (A) biot-2xm-Sav, (B) biot-2xm-S112Y-Sav, (C) biot-2xm-S112Y-K121Y-Sav, (D) biot-2xm-Sav + H_2O_2, (E) biot-2xm-S112Y-Sav + H_2O_2, and (F) biot-2xm-K121Y-Sav + H_2O_2. The chromatograms were integrated over this entire range, from 2.85 to 3.12 min, for all samples to capture the relative abundance of both the unoxidized and oxidized DF31. Mass spectra resulting from integration of this range are shown in figure S14.

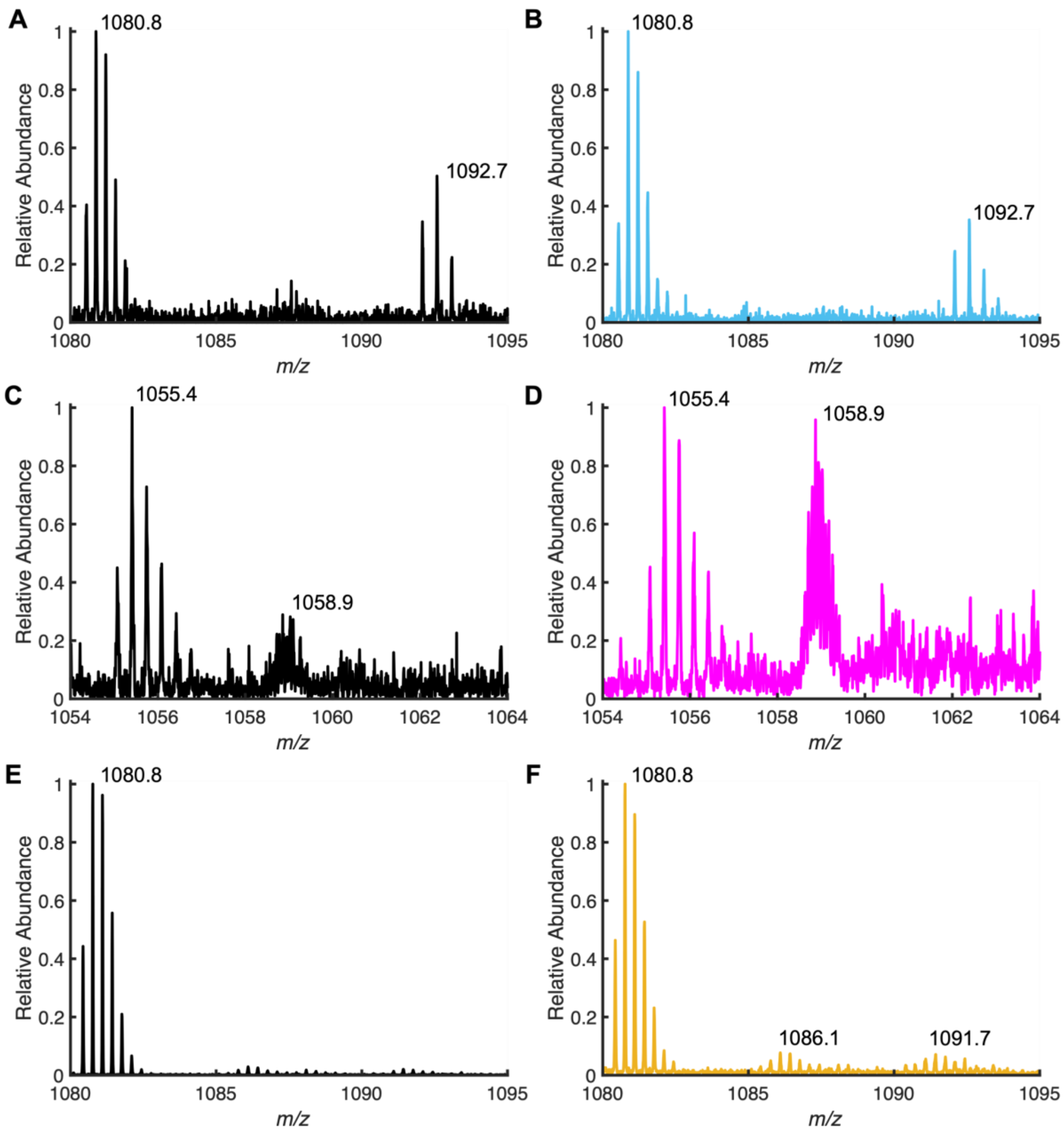

Figure S15. ESI-Q-TOF positive ion mode mass spectra of $[DF31]^{3+}$ resulting from tryptic digests: (A) biot-2xm-S112Y-Sav before and (B) after the addition of 10 equivalents of H_2O_2 and incubation for 1 h. (C) Biot-2xm-Sav before and (D) after the addition of 10 equivalents of H_2O_2 and incubation for 1 h. (E) Biot-2xm-K121Y-Sav before and (F) after the addition of 10 equivalents of H_2O_2 and incubation for 1 h.

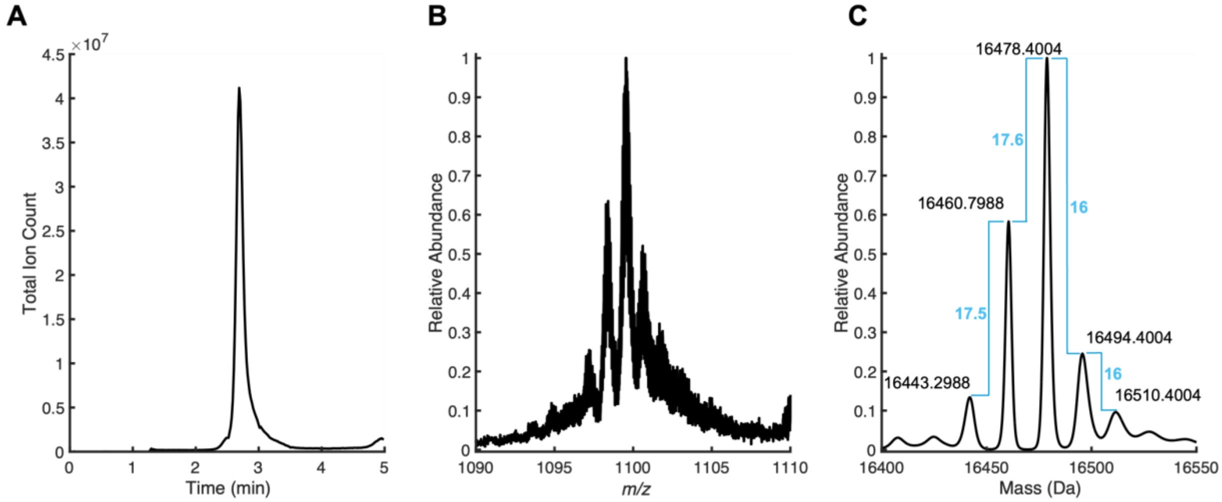

Figure S16. (A) Chromatogram of **1** incubated with $^{18}O_2H_2$. The major peak was integrated for further processing.

(B) ESI-Q-TOF positive ion mode mass spectra showing ions of highest abundance from integration of the major peaks of the chromatogram of **1** incubated with $^{18}O_2H_2$. The charge-state ion series containing these ions were used to reconstruct total average mass spectra shown in panel C.

(C) Deconvoluted ESI-Q-TOF positive ion mode mass spectrum of **1** incubated with $^{18}O_2H_2$. The nominal mass of 2xm-S112Y-Sav monomer is 16443.2998 Da. We observe two species that are consistent with the incorporation of ^{18}O derived from 90% isotope labeled $^{18}O_2H_2$. There are two more species consistent with nonspecific oxidation derived from ^{16}O.

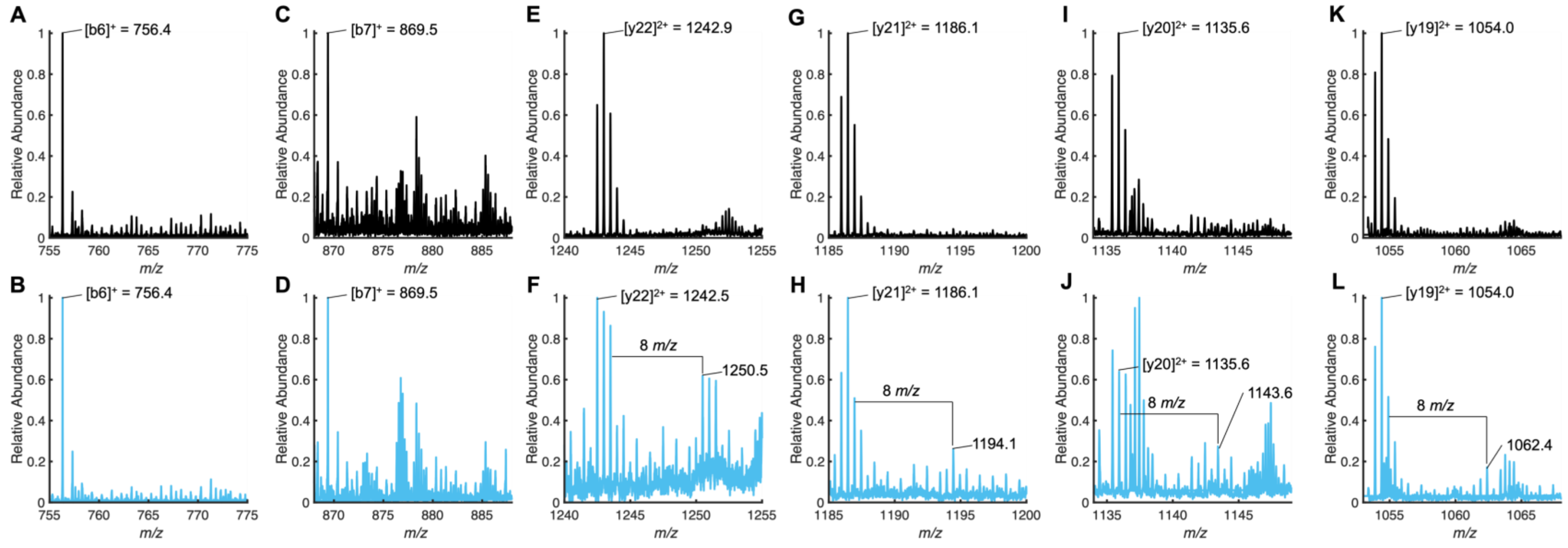

Figure S17. MS/MS analysis of b/y ion fragments of DF31 resulting from tryptic digestion o **1** indicating oxidation of residue Y112. Fragment b6 (A) before and (B) after incubation with H_2O_2, (C) and fragment b7 before and (D) after incubation with H_2O_2 are not oxidized, ruling out oxidation of residues 104-110. Fragment y22, y21, and y20 (E, G, I) before and (F, H, J) after incubation with H_2O_2. Oxidized fragments of y22, y21, and y20 appear after incubation with H_2O_2. (L) oxidation of W120 or H127 is also suggested by appearance of oxidized fragment y19 after incubation with H_2O_2 as y19 does not contain Y112.

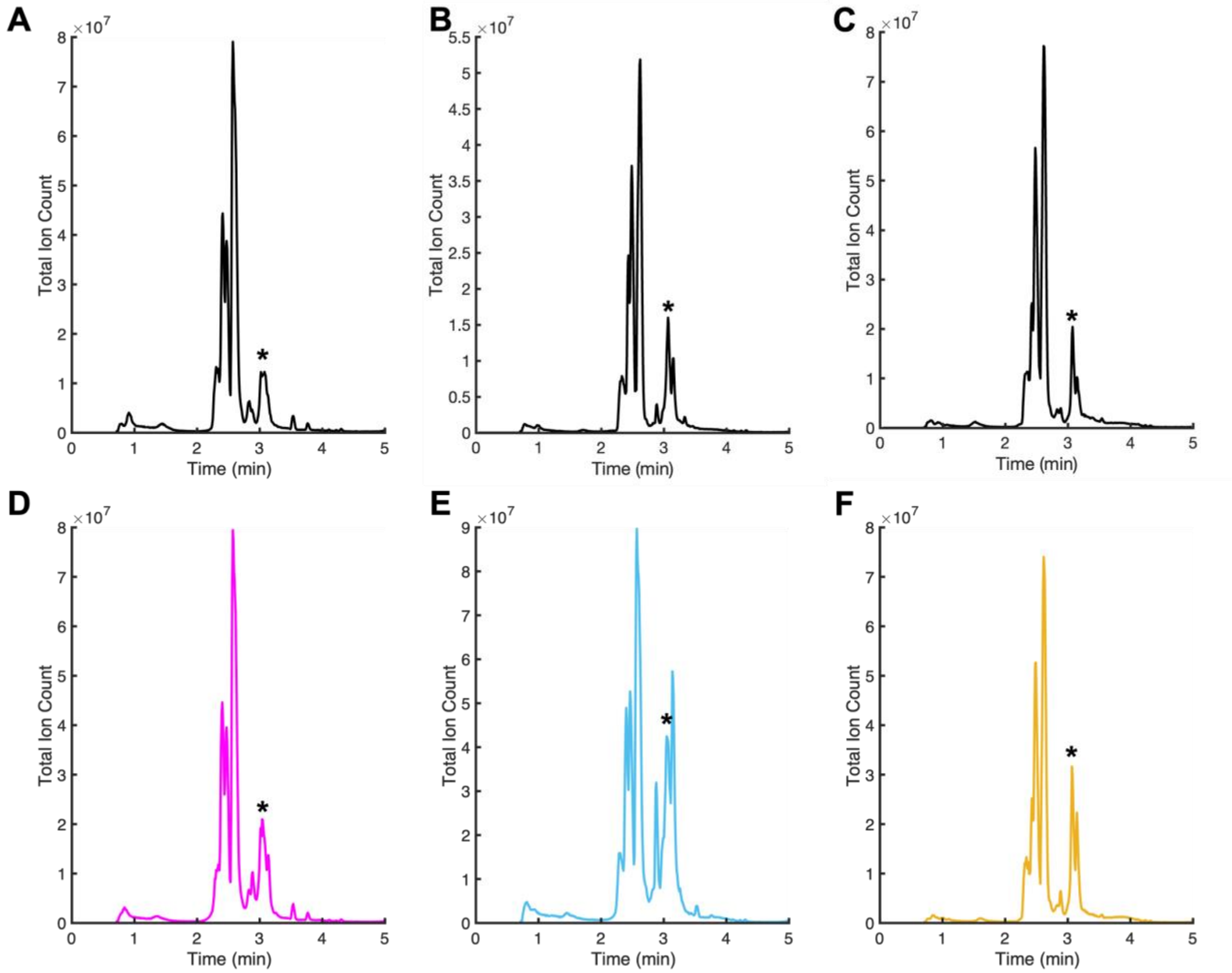

Figure S18. Chromatograms after trypsin digestion of (A) **2**, (B) **1**, (C) **3**, (D) **2** + H_2O_2, (E) **1** + H_2O_2, and (F) **3** + H_2O_2. Asterisks indicate chromatographic peaks containing DF31 in each sample.

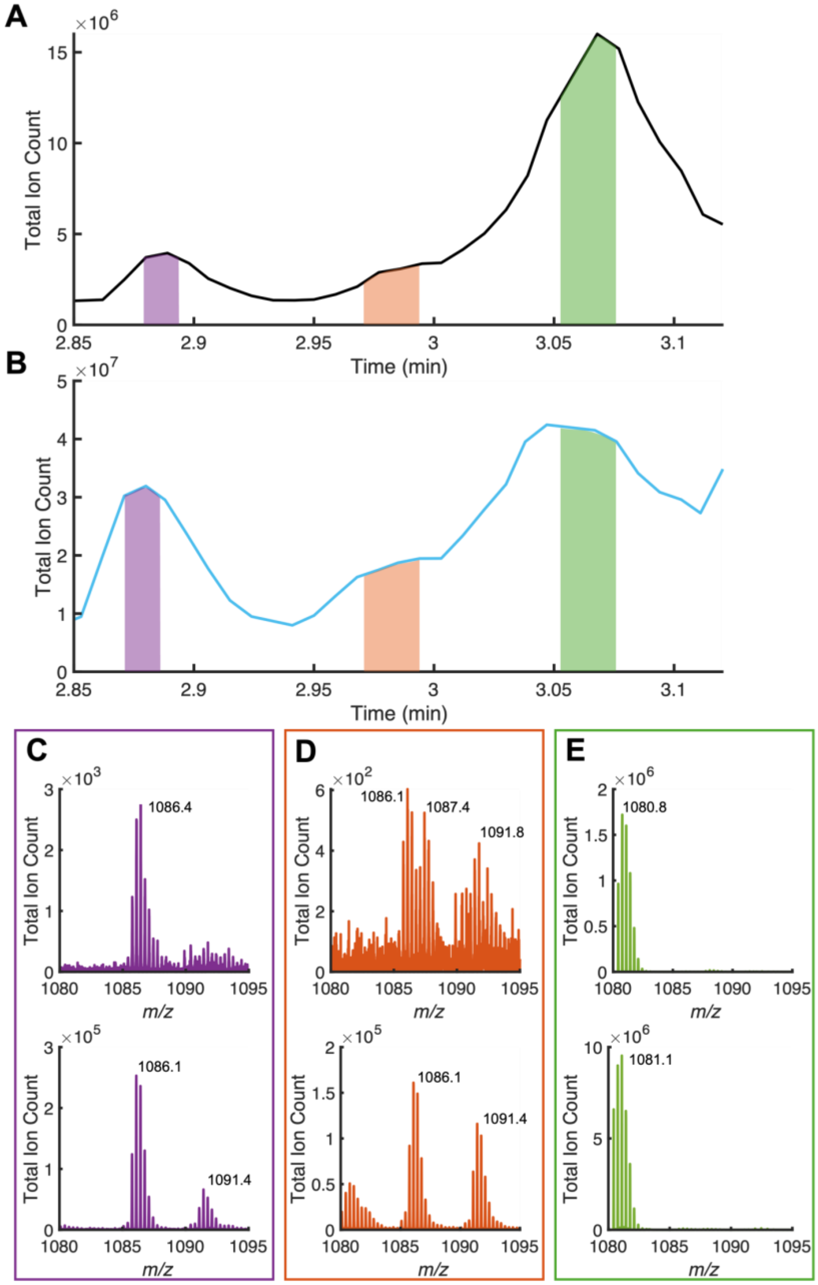

Figure S19. Total area of chromatograms integrated to produce mass spectra in Figure 4 for (A) **1** and (B) **1** + H_2O_2. Integrated area of chromatographic peak eluting at 2.87 min highlighted in purple to produce mass spectra in panel C for **1** (C, top) and **1** + 10 eq H_2O_2 (C, bottom). Integrated area of chromatographic peak eluting at 2.97 min highlighted in orange to produce mass spectra in panel D for **1** (D, top) and **1** + 10 eq H_2O_2 (D, bottom). Integrated area of chromatographic peak eluting at 3.06 min highlighted in green to produce mass spectra in panel E for **1** (E, top) and **1** + 10 eq H_2O_2 (E, bottom). Because of the presence of oxidized DF31 in **1** with and without H_2O_2 incubation (C and D top), the chromatograms were integrated over this entire range, from 2.85 to 3.12 min, for all samples to capture the relative abundance of both the unoxidized and oxidized DF31.

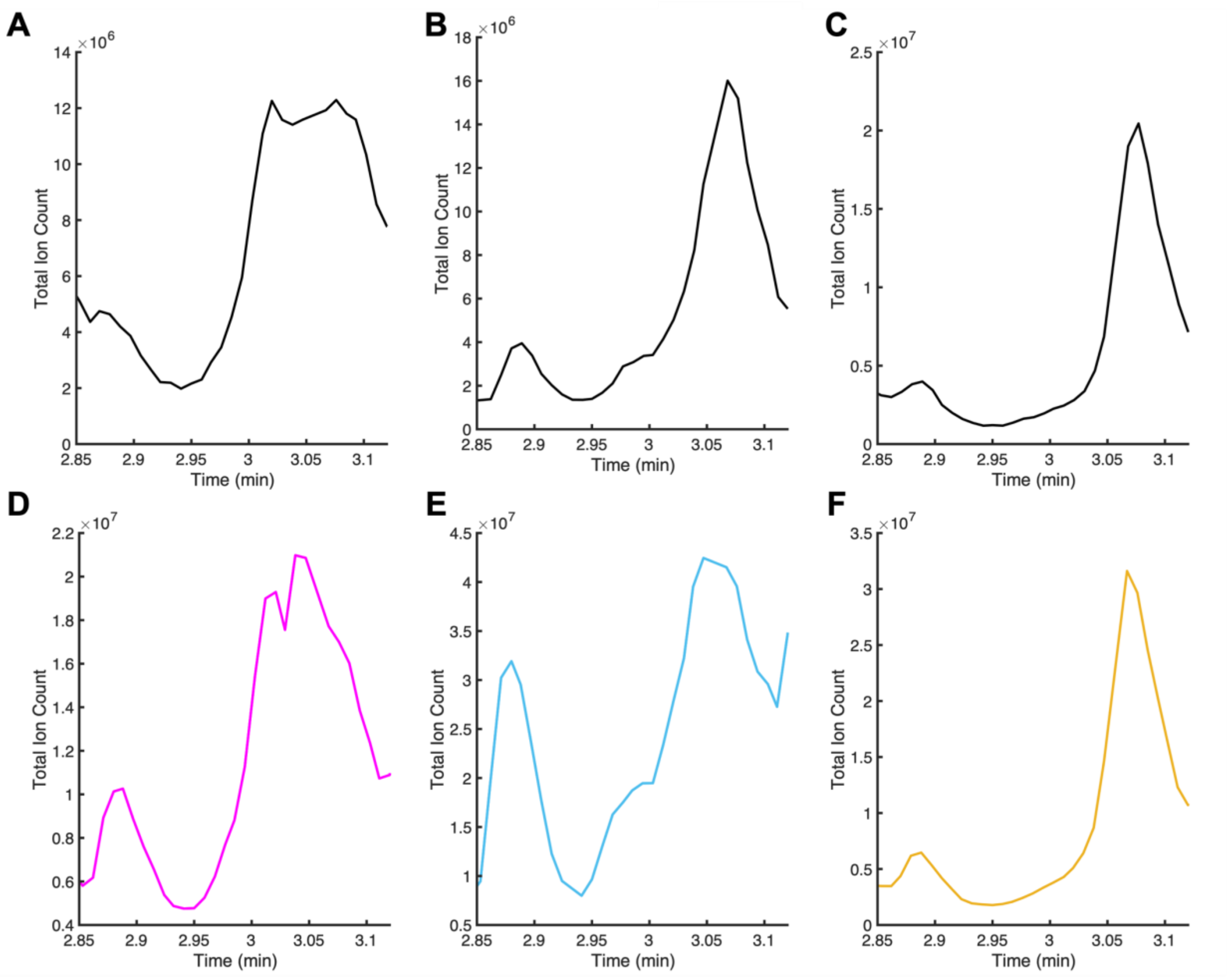

Figure S20. Integrated area of chromatogram after trypsin digestion of (A) **2**, (B) **1**, (C) **3**, (D) **2** + 10 eq H_2O_2, (E) **1** + 10 eq H_2O_2, and (F) **3** + 10 eq H_2O_2. Mass spectra resulting from integration of this area displayed in Figure 4.

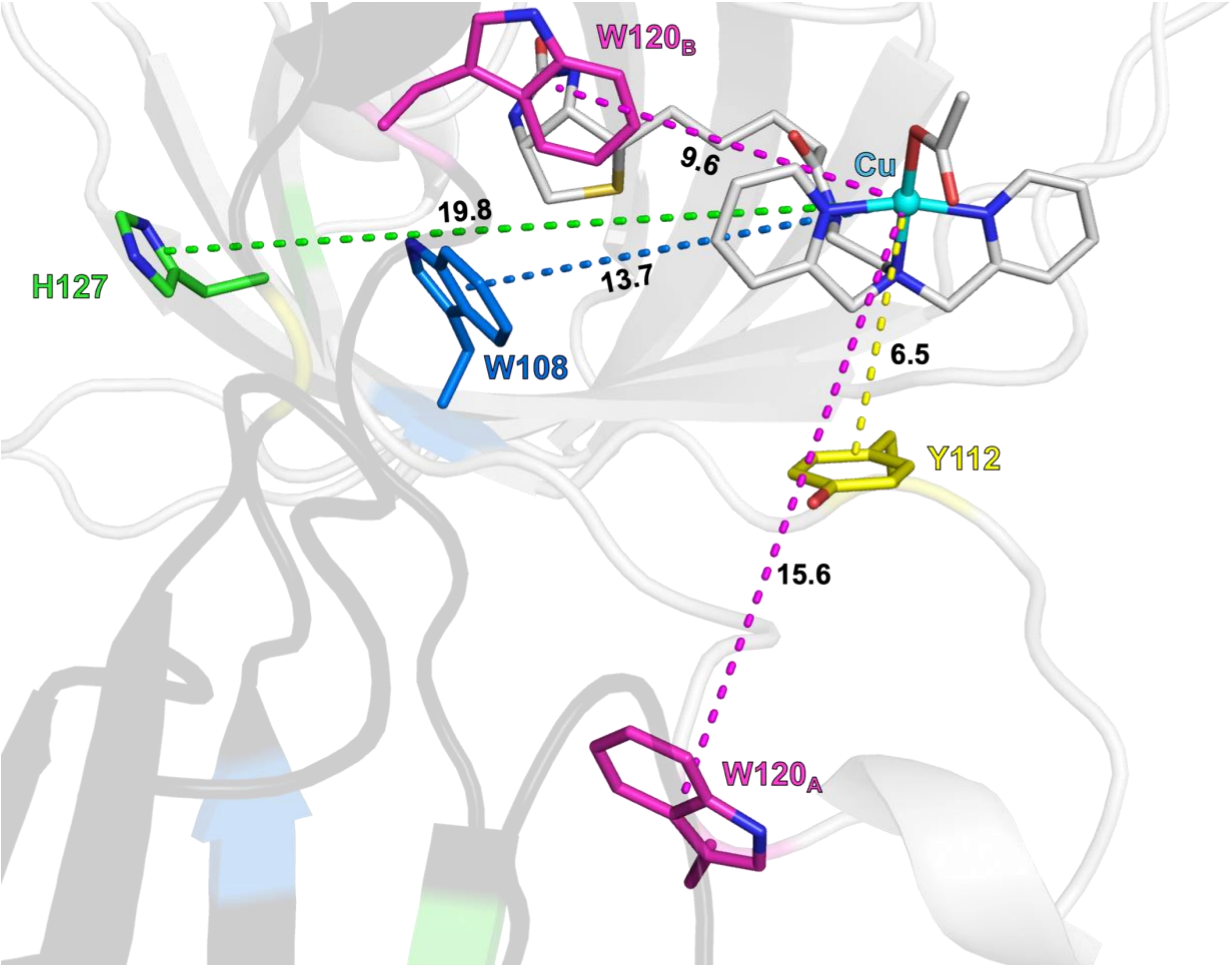

Figure S21. Molecular model of **1** (PDB: 9CSU). The protein is displayed as a cartoon and the Cu complex as well as residues W108, Y112, W120, and H127 are displayed as sticks. One monomeric subunit of Sav is shown in gray (deonted as A) and the adjacent symmetry related subunit (denoted as B) is shown in black. Residues in DF31 known to be susceptible to oxidation are displayed in blue, yellow, pink or green with the corresponding colored dashed lines indicating the distance from the copper center. Residue Y112 of the same subunit is the closest residue to the Cu center, making it most susceptible to Cu-mediated oxidation by H_2O_2.

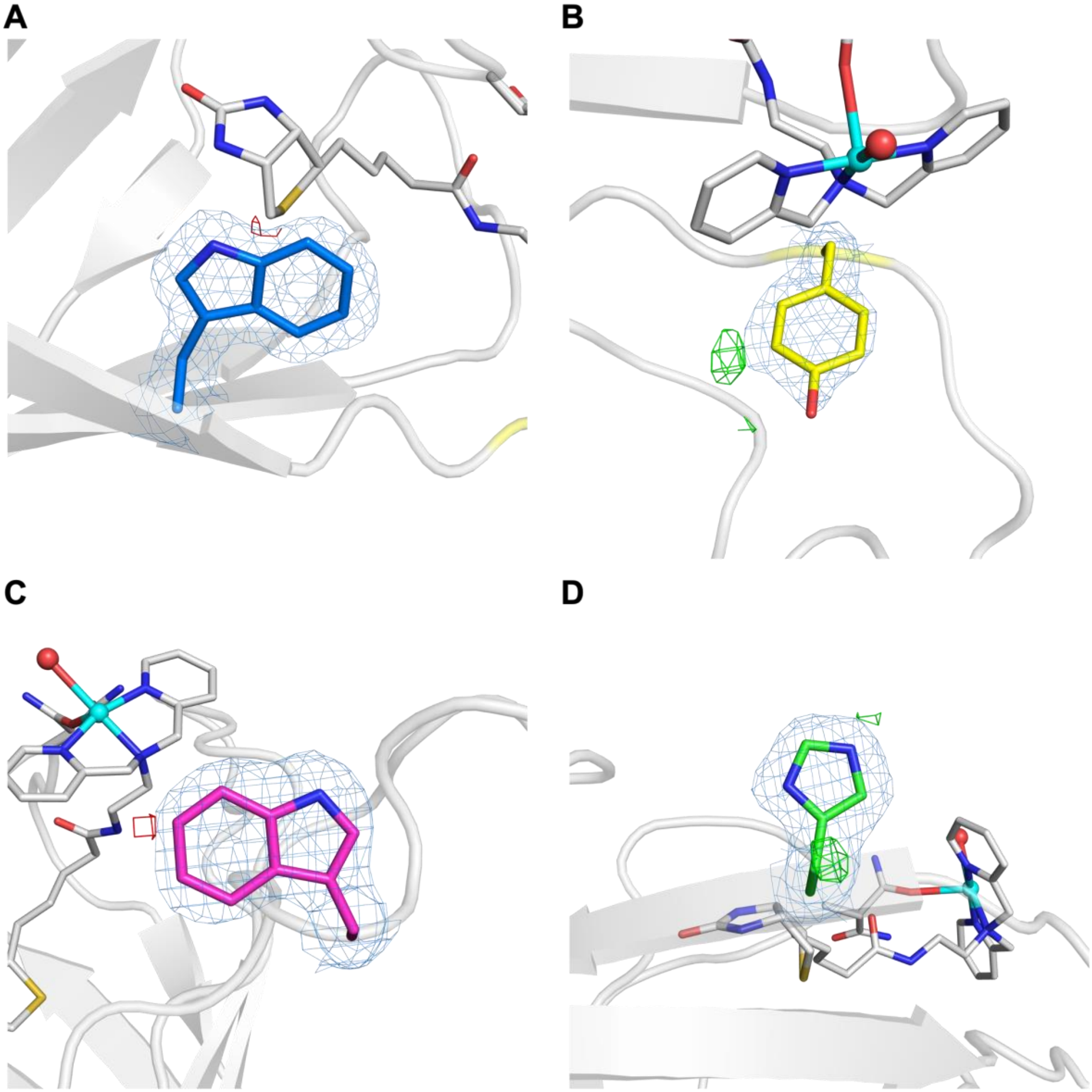

Figure S22. Molecular model of **1** + H_2O_2. The protein is displayed as a cartoon and Cu-cofactor as well as residues (A) W108, (B) Y112, (C) W120, and (D) H127 (residues of DF31 that may be susceptible to oxidation by H_2O_2) are displayed as sticks. Positive and negative difference density in F_o–F_c map around each residue contoured at 3σ shown in green and red mesh, respectively. The positions of the residues are indicated by the $2F_o$–F_c electron density shown in blue mesh contoured at 1σ.

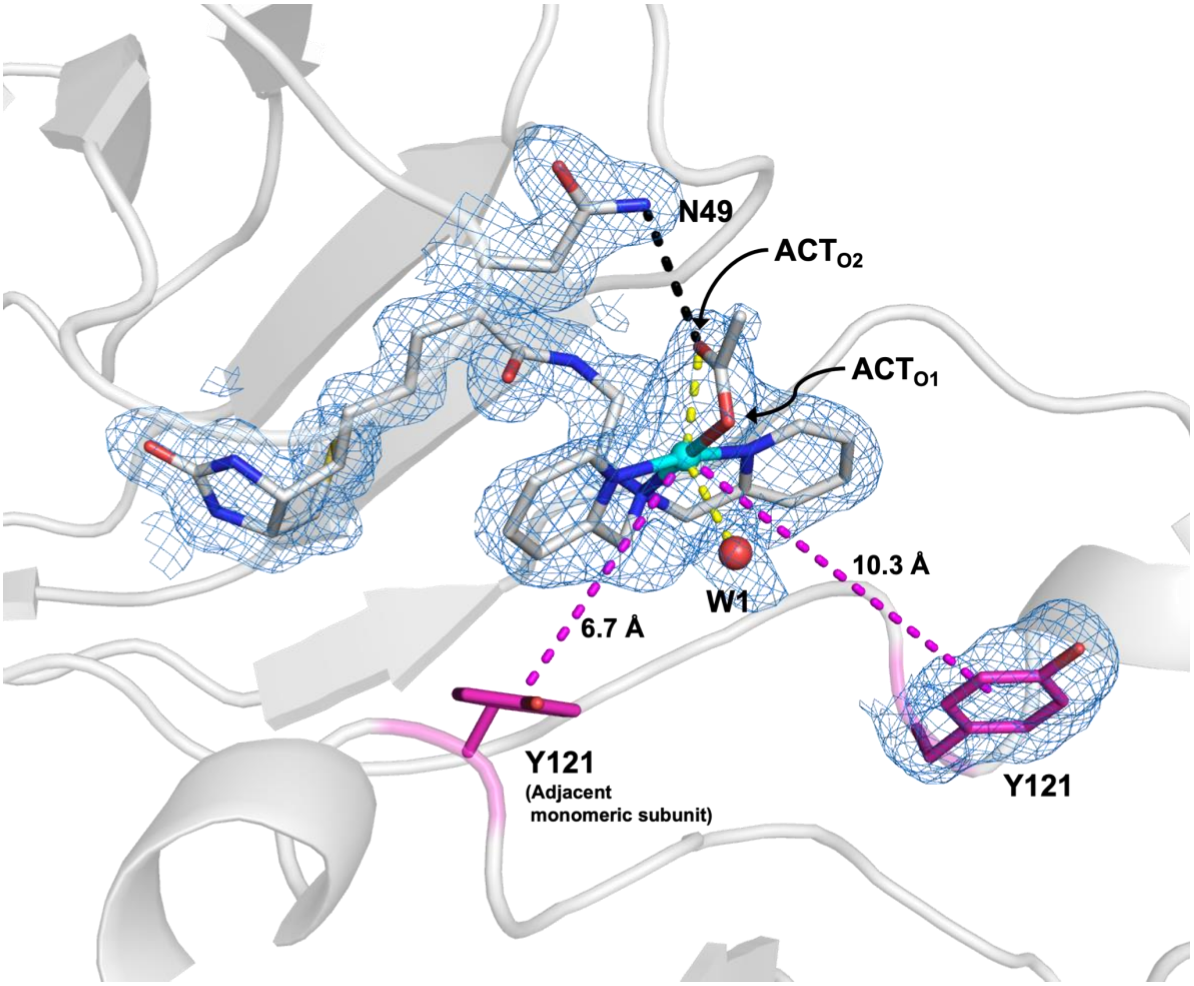

Figure S23. Molecular model of **3** (PDB: 9CSW). The protein is displayed in cartoon and the Cu complex as well as residues N49 and A121Y are displayed in sticks. Copper is colored in cyan and water molecules in red spheres. The position of the Cu-complex as well as residues N49 and Y121 are indicated by the $2F_o$-F_c electron density shown in blue mesh contoured at 1σ. Residue Y121 is shown in magenta and is 6.7 Å (adjacent monomeric Sav subunit) or 10.3 Å (same monomeric Sav subunit) from the Cu center where distances are indicated by magenta dashed lines. Hydrogen bonds are suggested in black dashed lines. The Cu-ACT$_{O1}$ and Cu-W1 distances are 2.5 and 2.6 Å, respectively, indicated by yellow dashed lines.

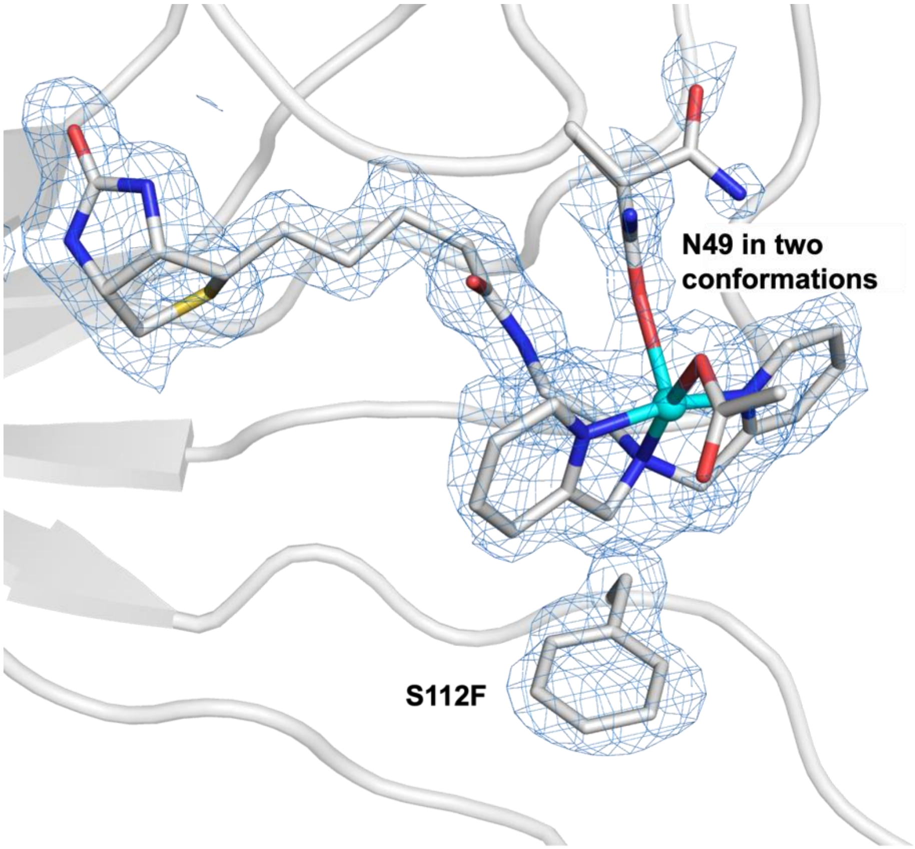

Figure S24. Molecular model of **4** (PDB: 9E6Z). The protein is displayed in cartoon and the Cu complex as well as residues N49 and S112F are displayed in sticks. Copper is colored in cyan and water molecules in red spheres. The position of the Cu-complex as well as residues N49 and F112 are indicated by the $2F_o$-F_c electron density shown in blue mesh contoured at 1σ.

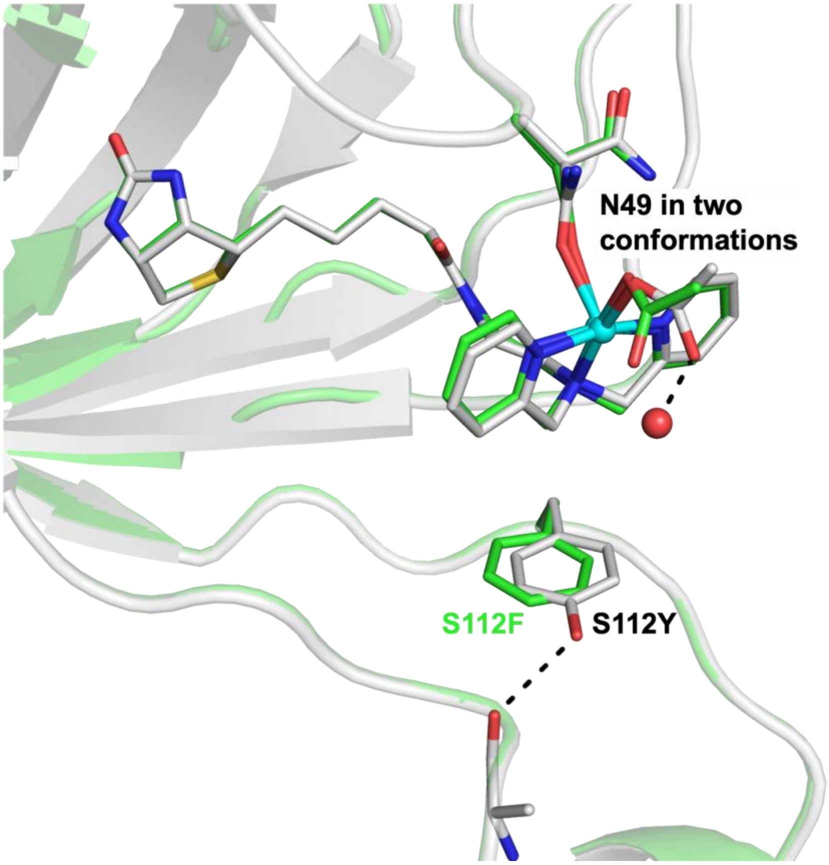

Figure S25. Molecular models of **1** (PDB: 9CSU, shown in gray) and **4** (PDB: 9E6Z, shown in green). The protein is displayed in cartoon and the Cu complex as well as residues N49 and S112Y/F are displayed in sticks. Copper is colored in cyan and water molecules in red spheres. Hydrogen bonds are suggested in black dashed lines. The orientation of S112Y and S112F relative to the Cu center are comparable, and both mutations support N49 coordination to the Cu cofactor.

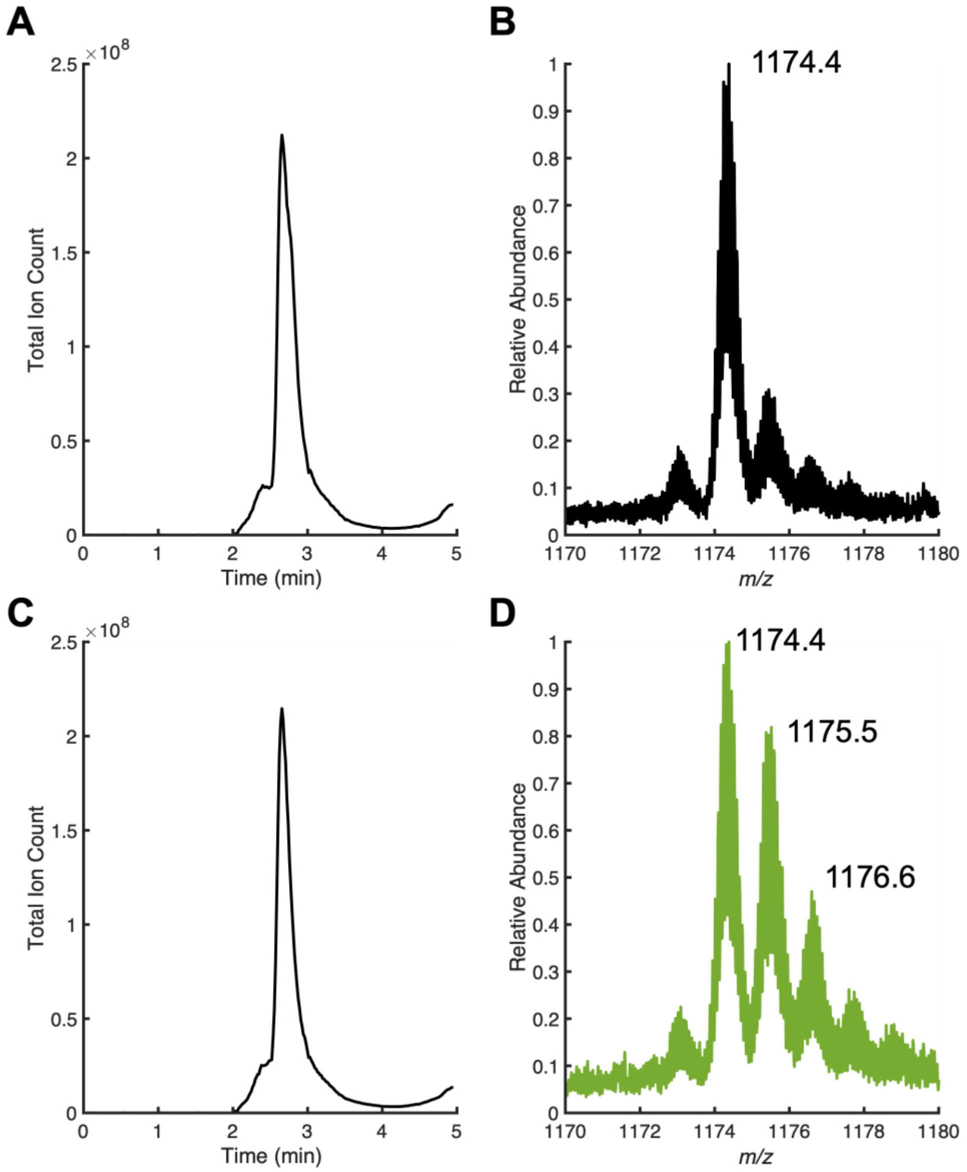

Figure S26. (A) Chromatogram of **4**. (B) ESI-Q-TOF positive ion mode mass spectra showing ions of highest abundance from integration of the major peak of the chromatogram in (A). (C) Chromatogram of **4** + H_2O_2. (D) ESI-Q-TOF positive ion mode mass spectra showing ions of highest abundance from integration of the major peak of the chromatogram in (C).

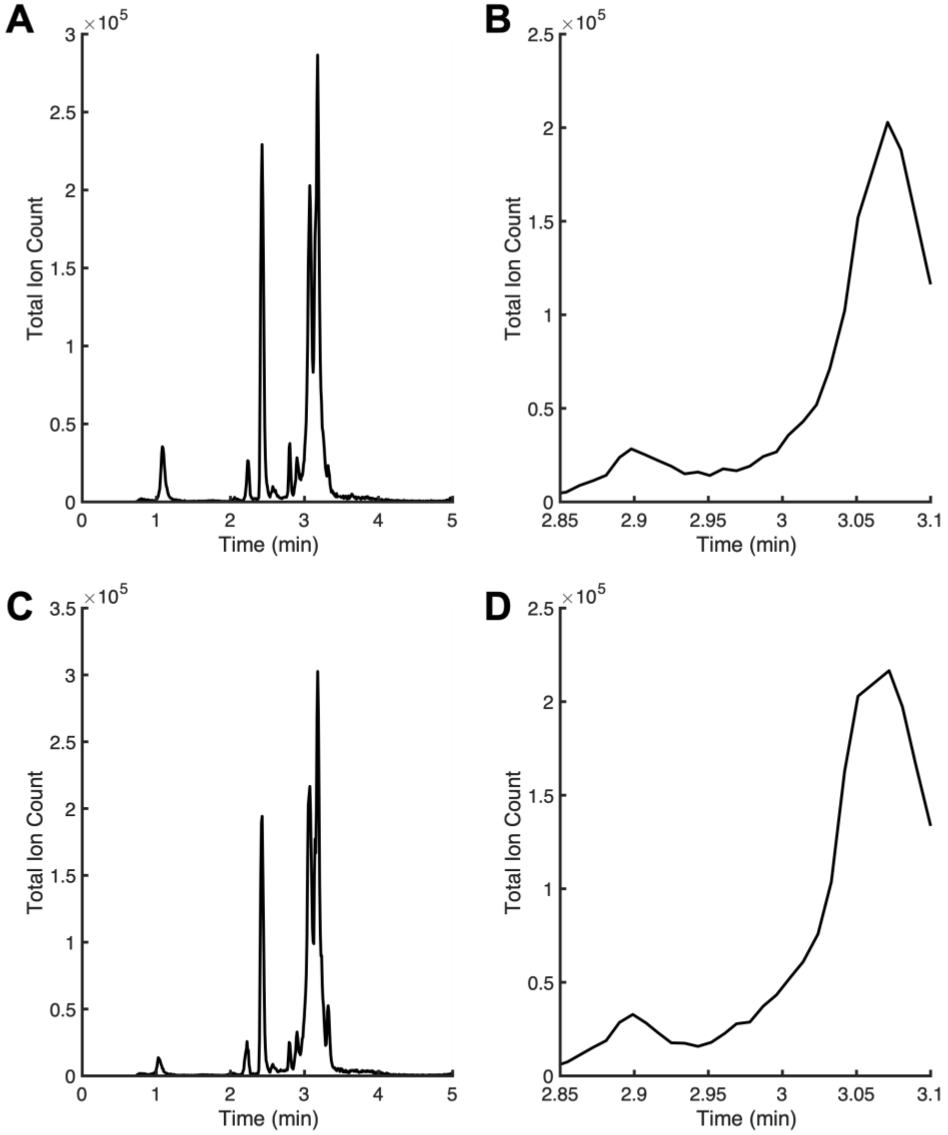

Figure S27. (A) Chromatogram of **4** after trypsin digestion. (B) Integrated area of chromatogram to produce Figure 4H. (C) Chromatogram of **4** + H_2O_2 after trypsin digestion. (C) Integrated area of chromatogram to produce Figure 4I.

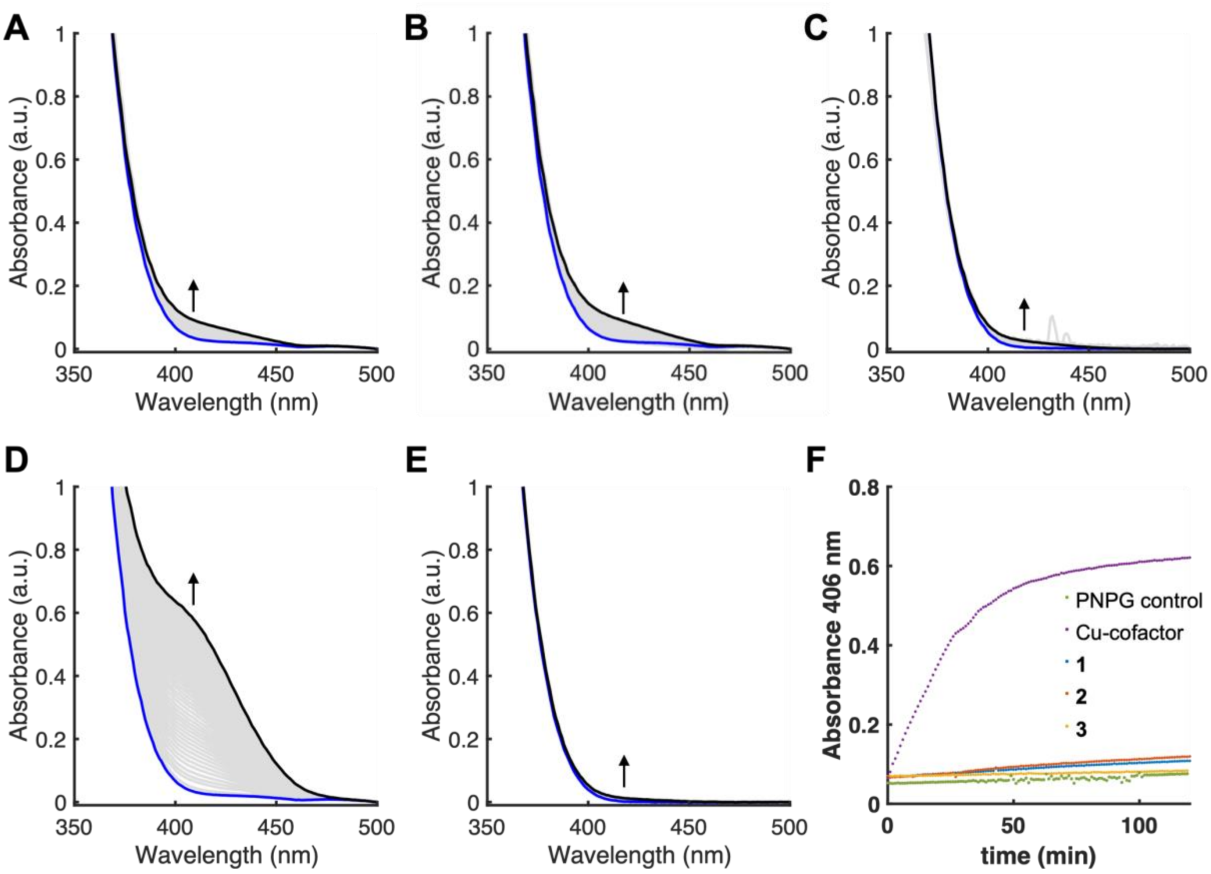

Figure S28. Reactivity of (A) **1**, (B) **2**, (C) **3** or (D) Cu cofactor with PNPG and H_2O_2 according to Scheme S2. Spectra of Cu-Sav with 200 equivalents of PNPG shown in black and after 120 min reaction with 200 equivalents of H_2O_2 shown in blue. (E) Reactivity of PNPG with H_2O_2 as a control. UV-vis absorbance at λ_{max} = 405 nm (corresponding to absorbance of 4-nitrophenolate) over 120 min incubation with H_2O_2.

Table S1. X-band EPR parameters for simulations shown in Figure S3. Due to anisotropic broadening, we cannot confidently assign A_2 or A_3 values.

		Cu-2xm-S112Y-Sav	Cu-2xm-S112Y-Sav + 10 H_2O_2 1 hr	Cu-2xm-S112Y-Sav + 10 H_2O_2 4 hr
g_1		2.23	2.23	2.23
g_2		2.08	2.07	2.07
g_3		2.04	2.04	2.04
A_1 (MHz)		500	495	501
A_2		85[a]	101[a]	91[a]
A_3		65[a]	62[a]	57[a]
Anisotropic Broadening (MHz)	A_1	224	220	217
	A_2	257	303	329
	A_3	143	145	139

[a] These values are approximations as they are smaller than the anisotropic broadening for their respective splittings.

Table S2. X-ray crystallography data processing and refinement statistics

**Sav Variant**	[Cu^{II}(biot-et-dpa)⊂2XM-Sav] (**2**)	[Cu^{II}(biot-et-dpa)⊂2XM-S112Y-Sav] (**1**)	[Cu^{II}(biot-et-dpa)⊂2XM-S112Y-Sav] + H_2O_2 (Y112 hydroxylated)	[Cu^{II}(biot-et-dpa)⊂2XM-S112Y-Sav] + H_2O_2 (2 Y112 rotamers)	[Cu^{II}(biot-et-dpa)⊂2XM-S112A-K121Y-Sav] (**3**)	[Cu^{II}(biot-et-dpa)⊂2XM-S112F-Sav] (**4**)
**PDB Code**	9CST	9CSU	9CSV	Not deposited	9CSW	9E6Z
**Data Processing**						
**Unit Cell (a, b, c, *α, β, γ*)**	57.82 Å, 57.82 Å, 184.14 Å, 90°, 90°, 90°	57.54 Å, 57.54 Å, 177.10 Å, 90°, 90°, 90°	57.55 Å, 57.55 Å, 177.73 Å, 90°, 90°, 90°		57.77 Å, 57.77 Å, 184.25 Å, 90°, 90°, 90°	57.54 Å, 57.54 Å, 176.28 Å, 90°, 90°, 90°
**Space Group**	$I4_122$	$I4_122$	$I4_122$		$I4_122$	$I4_122$
**Resolution (Å)**	42.09 – 1.13	54.72 – 1.60	27.38 – 1.60		42.08 – 1.302	41.11 – 1.7
**Highest resolution shell (Å)**	1.17 – 1.13	1.66 – 1.60	1.657 – 1.6		1.349 – 1.302	1.761 – 1.7
**R-merge (%)**	0.7592 (3.208)	0.7085 (4.619)	0.0964 (1.151)		0.6465 (3.806)	0.2381 (2.023)
**No. unique reflections**	58555 (5764)	20131 (1966)	20125 (1984)		38754 (3812)	16841 (1647)
**Multiplicity**	79.7 (79.9)	36.9 (37.1)	6.7 (6.9)		47.5 (48.2)	14.1 (14.5)
**Mean I/sig(I)**	60.09 (2.15)	13.47 (0.91)	9.00 (1.42)		62.91 (1.26)	8.98 (1.33)
**Completeness**	99.7 (99.88)	99.49 (97.31)	99.12 (99.90)		99.33 (99.08)	99.92 (100.00)
**CC(1/2)**	0.946 (0.784)	0.968 (0.309)	0.997 (0.561)		0.957 (0.607)	0.996 (0.524)
**Structure Refinement**						
**R-work**	0.1446 (0.2598)	0.1930 (0.3000)	0.2061 (0.3570)	0.2073 (0.3608)	0.1698 (0.3586)	0.2005 (0.3052)
**R-free**	0.1615 (0.2692)	0.2092 (0.3356)	0.2238 (0.3660)	0.2218 (0.3640)	0.1908 (0.3979)	0.2198 (0.3149)
**RMSD bond length (Å)**	0.014	0.004	0.007	0.008	0.008	0.014
**RMSD bond angle (°)**	1.29	0.66	0.88	0.88	1.00	1.27
**Avg B-factor (Å^2)**	20.10	25.01	30.25	30.24	22.91	20.26
**Protein**	18.28	23.64	29.25	29.25	21.75	19.50
**Cu Complex**	25.91	28.11	31.18	33.23	26.56	22.97
**Solvent**	31.85	39.77	39.99	39.34	32.37	28.46
**Occupancy**						
**Cu**	0.69, 0.31	0.65	0.74	0.74	0.63	0.78
**Acetate**	N/A	0.78	0.71	0.71	0.85	0.80
**N49**	1.00	0.61 (bound), 0.39	0.51 (bound), 0.49	0.51 (bound), 0.49	1.00	0.58 (bound), 0.42

Table S3. Select bond lengths and angles according to labeling in Figure S5.

	**Cu-2xm-S112Y-Sav (PDB: 9CSU)**	**Cu-2xm-S112Y-Sav + H_2O_2 (PDB: 9CSV)**
**Cu–N1**	1.9 Å	1.9 Å
**Cu–N2**	2.0 Å	2.1 Å
**Cu–N3**	2.1 Å	2.0 Å
**Cu–O3**	2.5 Å	2.4 Å
**Cu–O4$_{(ACT\ or\ W)}$**	2.4 Å	2.5 Å
**Cu–O5**	3.2 Å	N/A
**Cu–O6**	2.9 Å	N/A
**N1-Cu-N2**	83.2°	84.3°
**N2-Cu-N3**	82.0°	78.8°
**N3-Cu-O4$_{(ACT\ or\ W)}$**	99.5°	101.2°
**O4$_{(ACT\ or\ W)}$-Cu-O3**	87.6°	84.3°
**N1-Cu-O3**	94.2°	92.9°
**N2-Cu-O3**	101.7°	107.9°
**N3-Cu-O3**	93.1°	90.5°
**O1–O7**	3.0 Å	3.2 Å
**O2–O7**	N/A	2.8 Å
**O5–O6**	2.1 Å	N/A

Table S4. Intact protein LC-MS gradient method

**Time (mins)**	**Flow (mL/min)**	**% A**	**%B**	**Curve**
0	0.2	100	0	6
0.5	0.2	100	0	6
2	0.2	10	90	6
2.5	0.2	10	90	6
4	0.2	100	0	6
5	0.2	100	0	6

Table S5. Peptide LC-MS gradient method

**Time (mins)**	**Flow (mL/min)**	**% A**	**%B**	**Curve**
0	0.3	97	3	6
0.5	0.3	97	3	6
2	0.3	40	60	6
2.5	0.3	40	60	6
3	0.3	10	90	6
3.5	0.3	10	90	6
4	0.3	97	3	6
5	0.3	97	3	6

Table S6. Possible digest fragments of 2xm-S112Y-Sav. Identified ions [DF31]$^{3+}$ and [DF31]$^{2+}$ highlighted in yellow.

	mass	*m/z* +2 charge	*m/z* +3 charge	*m/z* +4 charge	*m/z* +5 charge	position	#MC	peptide sequence
1	12686.0661	6344.03305	4229.6887	3172.516525	2538.21322	12-131	5	DEAGITGTWYNQLGSTFIVT<br>AGADGALTGTYESAVGNAES<br>RYVLTGRYDSAPATDGSGTA<br>LGWTVAWKNNYRNAHSATTW<br>SGQYVGGAQARINTQWLLTY<br>GTTEANAWASTLVGHDTFTK
2	10569.9637	5285.98185	3524.321233	2643.490925	2114.99274	1-102	5	ASMTGGQQMGRDEAGITGTW<br>YNQLGSTFIVTAGADGALTG<br>TYESAVGNAESRYVLTGRYD<br>SAPATDGSGTALGWTVAWKN<br>NYRNAHSATTWSGQYVGGAQ AR
3	9692.771	4847.3855	3231.923667	2424.19275	1939.5542	53-143	5	YVLTGRYDSAPATDGSGTAL<br>GWTVAWKNNYRNAHSATTWS<br>GQYVGGAQARINTQWLLTYG<br>TTEANAWASTLVGHDTFTKV KPSAASIDAAK
4	9465.4832	4733.7416	3156.161067	2367.3708	1894.09664	12-102	4	DEAGITGTWYNQLGSTFIVT<br>AGADGALTGTYESAVGNAES<br>RYVLTGRYDSAPATDGSGTA<br>LGWTVAWKNNYRNAHSATTW SGQYVGGAQAR
5	9131.4799	4566.73995	3044.826633	2283.869975	1827.29598	59-144	5	YDSAPATDGSGTALGWTVAW<br>KNNYRNAHSATTWSGQYVGG<br>AQARINTQWLLTYGTTEANA<br>WASTLVGHDTFTKVKPSAAS IDAAKK
6	9003.3849	4502.69245	3002.1283	2251.846225	1801.67698	59-143	4	YDSAPATDGSGTALGWTVAW<br>KNNYRNAHSATTWSGQYVGG<br>AQARINTQWLLTYGTTEANA<br>WASTLVGHDTFTKVKPSAAS IDAAK
7	8627.0603	4314.53015	2876.686767	2157.765075	1726.41206	1-83	4	ASMTGGQQMGRDEAGITGTW<br>YNQLGSTFIVTAGADGALTG<br>TYESAVGNAESRYVLTGRYD<br>SAPATDGSGTALGWTVAWKN NYR
8	8554.1364	4278.0682	2852.3788	2139.5341	1711.82728	53-131	4	YVLTGRYDSAPATDGSGTAL<br>GWTVAWKNNYRNAHSATTWS<br>GQYVGGAQARINTQWLLTYG<br>TTEANAWASTLVGHDTFTK
9	8374.1476	4188.0738	2792.382533	2094.5369	1675.82952	80-158	5	NNYRNAHSATTWSGQYVGGA<br>QARINTQWLLTYGTTEANAW<br>ASTLVGHDTFTKVKPSAASI<br>DAAKKAGVNNGNPLDAVQQ
10	8079.81	4040.905	2694.27	2020.9525	1616.962	1-79	3	ASMTGGQQMGRDEAGITGTW<br>YNQLGSTFIVTAGADGALTG<br>TYESAVGNAESRYVLTGRYD<br>SAPATDGSGTALGWTVAWK
11	7864.7504	3933.3752	2622.583467	1967.1876	1573.95008	59-131	3	YDSAPATDGSGTALGWTVAW<br>KNNYRNAHSATTWSGQYVGG<br>AQARINTQWLLTYGTTEANA WASTLVGHDTFTK
12	7826.8973	3914.44865	2609.965767	1957.724325	1566.37946	84-158	4	NAHSATTWSGQYVGGAQARI<br>NTQWLLTYGTTEANAWASTL<br>VGHDTFTKVKPSAASIDAAK<br>KAGVNNGNPLDAVQQ
13	7522.5798	3762.2899	2508.5266	1881.64495	1505.51596	12-83	3	DEAGITGTWYNQLGSTFIVT<br>AGADGALTGTYESAVGNAES<br>RYVLTGRYDSAPATDGSGTA LGWTVAWKNNYR
14	6996.484	3499.242	2333.161333	1750.121	1400.2968	80-144	4	NNYRNAHSATTWSGQYVGGA<br>QARINTQWLLTYGTTEANAW<br>ASTLVGHDTFTKVKPSAASI DAAKK
15	6975.3295	3488.66475	2326.109833	1744.832375	1396.0659	12-79	2	DEAGITGTWYNQLGSTFIVT<br>AGADGALTGTYESAVGNAES<br>RYVLTGRYDSAPATDGSGTA LGWTVAWK
16	6868.389	3435.1945	2290.463	1718.09725	1374.6778	80-143	3	NNYRNAHSATTWSGQYVGGA<br>QARINTQWLLTYGTTEANAW<br>ASTLVGHDTFTKVKPSAASI DAAK

17	6449.2337	3225.61685	2150.744567	1613.308425	1290.84674	84-144	3	NAHSATTWSGQYVGGAQARI NTQWLLTYGTTEANAWASTL VGHDTFTKVKPSAASIDAAK K
18	6321.1387	3161.56935	2108.046233	1581.284675	1265.22774	84-143	2	NAHSATTWSGQYVGGAQARI NTQWLLTYGTTEANAWASTL VGHDTFTKVKPSAASIDAAK
19	5944.814	2973.407	1982.604667	1487.2035	1189.9628	1-58	2	ASMTGGQQMGRDEAGITGTW YNQLGSTFIVTAGADGALTG TYESAVGNAESRYVLTGR
20	5883.9939	2942.99695	1962.3313	1471.998475	1177.79878	103-158	3	INTQWLLTYGTTEANAWAST LVGHDTFTKVKPSAASIDAA KKAGVNNGNPLDAVQQ
21	5729.7544	2865.8772	1910.918133	1433.4386	1146.95088	80-131	2	NNYRNAHSATTWSGQYVGGA QARINTQWLLTYGTTEANAW ASTLVGHDTFTK
22	5333.5535	2667.77675	1778.851167	1334.388375	1067.7107	53-102	3	YVLTGRYDSAPATDGSGTAL GWTVAWKNNYRNAHSATTWS GQYVGGAQAR
23	5255.428	2628.714	1752.809333	1314.857	1052.0856	1-52	1	ASMTGGQQMGRDEAGITGTW YNQLGSTFIVTAGADGALTG TYESAVGNAESR
24	5182.5041	2592.25205	1728.501367	1296.626025	1037.50082	84-131	1	NAHSATTWSGQYVGGAQARI NTQWLLTYGTTEANAWASTL VGHDTFTK
25	4840.3336	2421.1668	1614.444533	1211.0834	969.06672	12-58	1	DEAGITGTWYNQLGSTFIVT AGADGALTGTYESAVGNAES RYVLTGR
26	4644.1675	2323.08375	1549.055833	1162.041875	929.8335	59-102	2	YDSAPATDGSGTALGWTVAW KNNYRNAHSATTWSGQYVGG AQAR
27	4506.3303	2254.16515	1503.1101	1127.582575	902.26606	103-144	2	INTQWLLTYGTTEANAWAST LVGHDTFTKVKPSAASIDAA KK
28	4378.2353	2190.11765	1460.411767	1095.558825	876.64706	103-143	1	INTQWLLTYGTTEANAWAST LVGHDTFTKVKPSAASIDAA K
29	4150.9475	2076.47375	1384.649167	1038.736875	831.1895	12-52	0	DEAGITGTWYNQLGSTFIVT AGADGALTGTYESAVGNAES R
30	3390.6501	1696.32505	1131.2167	848.662525	679.13002	53-83	2	YVLTGRYDSAPATDGSGTAL GWTVAWKNNYR
31	3239.6007	1620.80035	1080.8669	810.900175	648.92014	103-131	0	INTQWLLTYGTTEANAWAST LVGHDTFTK
32	2843.3998	1422.6999	948.7999333	711.84995	569.67996	53-79	1	YVLTGRYDSAPATDGSGTAL GWTVAWK
33	2701.2641	1351.63205	901.4213667	676.316025	541.25282	59-83	1	YDSAPATDGSGTALGWTVAW KNNYR
34	2663.411	1332.7055	888.8036667	666.85275	533.6822	132-158	2	VKPSAASIDAAKKAGVNNGN PLDAVQQ
35	2509.1715	1255.58575	837.3905	628.292875	502.8343	80-102	1	NNYRNAHSATTWSGQYVGGA QAR
36	2154.0138	1078.0069	719.0046	539.50345	431.80276	59-79	0	YDSAPATDGSGTALGWTVAW K
37	1961.9212	981.9606	654.9737333	491.4803	393.38424	84-102	0	NAHSATTWSGQYVGGAQAR
38	1524.7765	763.38825	509.2588333	382.194125	305.9553	144-158	1	KAGVNNGNPLDAVQQ
39	1396.6815	699.34075	466.5605	350.170375	280.3363	145-158	0	AGVNNGNPLDAVQQ
40	1285.7474	643.8737	429.5824667	322.43685	258.14948	132-144	1	VKPSAASIDAAKK
41	1157.6524	579.8262	386.8841333	290.4131	232.53048	132-143	0	VKPSAASIDAAK
42	1123.4983	562.74915	375.4994333	281.874575	225.69966	1-11	0	ASMTGGQQMGR
43	708.4039	355.20195	237.1346333	178.100975	142.68078	53-58	0	YVLTGR
44	566.2681	284.13405	189.7560333	142.567025	114.25362	80-83	0	NNYR

Table S7. Calculated b/y ion fragments of DF31 in 2xm-S112Y-Sav. Identified unmodified fragments highlighted in yellow. Identified oxidized fragments highlighted in blue.

b++	b+	b#	Residue	y#	y+	y++
--	--	1	I	28	--	--
114.5708	228.1343	2	N	27	2998.4217	1563.7620
165.0946	329.1819	3	T	26	2884.3788	1506.7405
229.1239	457.2405	4	Q	25	2783.3311	1456.2167
322.1636	643.3198	5	W	24	2655.2726	1392.1874
378.7056	756.4039	6	L	23	2469.1932	1299.1477
435.2476	869.4880	7	L	22	2356.1092	1242.6057
485.7715	970.5356	8	T	21	2243.0251	1186.0637
567.3031	1133.5990	9	Y	20	2141.9774	1135.5398
595.8139	1190.6204	10	G	19	1978.9141	1054.0082
646.3377	1291.6681	11	T	18	1921.8926	1025.4974
696.8615	1392.7158	12	T	17	1820.8450	974.9736
761.3828	1521.7584	13	E	16	1719.7973	924.4498
796.9014	1592.7955	14	A	15	1590.7547	859.9285
853.9229	1706.8384	15	N	14	1519.7176	824.4099
889.4414	1777.8755	16	A	13	1405.6747	767.3884
982.4811	1963.9549	17	W	12	1334.6375	731.8699
1017.9996	2034.9920	18	A	11	1148.5582	638.8302
1061.5156	2122.0240	19	S	10	1077.5211	603.3117
1112.0395	2223.0717	20	T	9	990.4891	559.7957
1168.5815	2336.1557	21	L	8	889.4414	509.2718
1218.1157	2435.2242	22	V	7	776.3573	452.7298
1246.6264	2492.2456	23	G	6	677.2889	403.1956
1315.1559	2629.3045	24	H	5	620.2675	374.6849
1372.6694	2744.3315	25	D	4	483.2086	306.1554
1423.1932	2845.3791	26	T	3	368.1816	248.6419
1496.7274	2992.4476	27	F	2	267.1339	198.1180
--	--	28	T	1	120.0655	124.5839

References

1 A. B. Pangborn, M. A. Giardello, R. H. Grubbs, R. K. Rosen, F. J. Timmers and M. Beckman, *Oranometallics*, 1996, 15, 1518–1520.

2 J. M. Chambers, L. M. Lindqvist, A. Webb, D. C. S. Huang, G. P. Savage and M. A. Rizzacasa, *Org Lett*, 2013, 15, 1406–1409.

3 S. A. Leaver, M. Palaniandavar, C. A. Kilner and M. A. Halcrow, *Journal of the Chemical Society. Dalton Transactions*, 2003, 3, 4224–4225.

4 K. R. Miller, J. D. Paretsky, A. H. Follmer, T. Heinisch, K. Mittra, S. Gul, I. S. Kim, F. D. Fuller, A. Batyuk, K. D. Sutherlin, A. S. Brewster, A. Bhowmick, N. K. Sauter, J. Kern, J. Yano, M. T. Green, T. R. Ward and A. S. Borovik, *Inorg Chem*, 2020, 59, 6000–6009.

5 H. Mallin, M. Hestericová, R. Reuter and T. R. Ward, *Nat Protoc*, 2016, 11, 835–852.

6 K. R. Miller, S. Biswas, A. Jasniewski, A. H. Follmer, A. Biswas, T. Albert, S. Sabuncu, E. L. Bominaar, M. P. Hendrich, P. Moënne-Loccoz and A. S. Borovik, *J Am Chem Soc*, 2021, 143, 2384–2393.

7 S. I. Mann, T. Heinisch, T. R. Ward and A. S. Borovik, *J Am Chem Soc*, 2017, 139, 17289–17292.

8 P. Dixit, B. Basu, M. Puri, D. K. Tuli, A. S. Mathur and C. J. Barrow, *Biotechnol Biofuels*, , DOI:10.1186/s13068-019-1526-4.

9 T. G. G. Battye, L. Kontogiannis, O. Johnson, H. R. Powell and A. G. W. Leslie, *Acta Crystallogr D Biol Crystallogr*, 2011, 67, 271–281.

10 W. Kabsch, *Acta Crystallogr D Biol Crystallogr*, 2010, 66, 125–132.

11 P. R. Evans, *Acta Crystallogr D Biol Crystallogr*, 2011, 67, 282–292.

12 P. D. Adams, P. V. Afonine, G. Bunkóczi, V. B. Chen, I. W. Davis, N. Echols, J. J. Headd, L. W. Hung, G. J. Kapral, R. W. Grosse-Kunstleve, A. J. McCoy, N. W. Moriarty, R. Oeffner, R. J. Read, D. C. Richardson, J. S. Richardson, T. C. Terwilliger and P. H. Zwart, *Acta Crystallogr D Biol Crystallogr*, 2010, 66, 213–221.

13 G. N. Murshudov Alexe, A. Vagin and E. J. Dodson, *Refinement of Macromolecular Structures by the Maximum-Likelihood Method*, 1997, vol. 53.

14 P. Emsley and K. Cowtan, *Acta Crystallogr D Biol Crystallogr*, 2004, 60, 2126–2132.

15 C. W. Koo, F. J. Tucci, Y. He and A. C. Rosenzweig, *Recovery of particulate methane monooxygenase structure and activity in a lipid bilayer*, .

16 P. J. Hart, A. M. Nersissian, R. G. Herrmann, R. M. Nalbandyan, J. S. Valentine and D. Eisenberg, *Protein Science*, 1996, 5, 2175–2183.

17 J. C. Cottrell and B. N. Green, *MaxEnt: An Essential Maximum Entropy Based Tool for Interpreting Multiply-Charged Electrospray Data*, .
